# Regional variability in reproductive traits of the *Acropora hyacinthus* species complex in the Western Pacific Region

**DOI:** 10.1371/journal.pone.0208605

**Published:** 2019-01-29

**Authors:** Adriana Maria Santacruz-Castro

**Affiliations:** Department of Life Science, National Taiwan Normal University and and Biodiversity Program, Taiwan International Graduate Program, Academia Sinica, Taipei, Taiwan; Fred Hutchinson Cancer Research Center, UNITED STATES

## Abstract

Understanding natural variations in the life history traits of reef-building corals under different environmental conditions is an area of active research. This study compares variability in the reproductive and genetic traits of the hermaphroditic broadcast spawning coral *Acropora hyacinthus*, from the Western Pacific Region, across six different latitudes [Japan (33° and 31°N), Taiwan (23°, 22° and 21°N), and Indonesia (5°S)]. Egg sizes among corals in the lowest latitude studied were significantly larger than those at high latitudes, while the mean number of eggs were significantly different only among high latitude and two out of the three mid latitude locations studied. Egg numbers were significantly negatively correlated with egg and testis volumes, indicating reproductive trade-offs across locations. Female gonad volumes were smaller at high latitudes but significantly larger at lower latitudes, being positively correlated with seawater temperatures. Furthermore, high genetic similarities among populations suggest active gene flow among low-, mid- and high-latitude locations. An exception to this trend, the mid-latitude location of Penghu (off western Taiwan) formed an independent group with highly similar genetic and reproductive traits, suggesting reproductive isolation with local adaptations. This study reports natural spatial variations in the reproductive traits of *A*. *hyacinthus* at different latitudinal locations, which may serve as baseline information to predict how the life histories of corals in general respond to the impacts of climate change.

## Introduction

Several aquatic animals present variations in life-history strategies within and among populations [1]. For example, egg size and number (fecundity) are known to be inversely correlated. Additionally, due to differences in female size, resource availability or spawning season, egg size may vary within populations [2]. There is also a general trend within and among species, known as the “Thorson-Rass” rule, in which egg size increases with latitude [3].

Reef-building corals show intraspecific variation along a latitudinal gradient in the timing of their annual spawning, with a delay in coral spawning that is correlated to lower mean seawater temperatures at higher latitudes [4, 5]. There is also variation in the reproductive traits of corals species among geographic populations [6], among genus members [7, 8], among cohorts within the reproductive cycle of the same species [9, 10], as well as within colonies (i.e., inter-polyp variations) [11, 12]. However, it is still unclear if these variations are related to local environmental pressures (e.g., local temperature and light irradiance variations) or if the populations have genetically diverged due to either geographic or reproductive isolation.

These questions remain to be studied for most Scleractinian species. Corals such as *Pocillopora verrucosa* [13] and *Echinopora lamellosa* [14] from the Indo-Pacific present larger egg sizes at higher latitudes compared to tropical populations. This behavior suggests increased energy investment towards larval survivorship as a response to the unfavorable environmental conditions at higher latitudes. By contrast, octocorals present smaller egg sizes at higher latitudes compared to tropical locations, which has been attributed to differences in the maternal effects at different latitudes [15]. For example, mature oocytes of the octocoral *Dendronephthya hemprichi* are from 260 μm to 500 μm south of Eliat (~28°N) [16], whereas other octocorals, including *Anthelia glauca*, *Sarcophyton laucum*, *S*. *elegans* and *Lobophytum pauciflorum*, produce larger mature oocytes (>500 μm) in tropical and subtropical regions [17–19].

*Acropora* is the most diverse genus and one of the most abundant reef-building corals in the Indo-Pacific [20]. This genus is known for its high levels of genetic polymorphism between species that are influenced by widespread genetic exchange through introgression, resulting in multiple cross-fertilizable cryptic and sister species [21–23]. *Acropora hyacinthus* has an extensive geographic distribution [20] and different colony morphs [24], and has been identified as part of a large syngameon (a complex network of gene flow among species) with multiple sympatric and allopatric cryptic species occurring in the Indo-Pacific [23] and Kuroshio Region [21], serving as a good model for the study of spatial variations.

Major spawning events have been reported to occur for *A*. *hyacinthus* in the Western Pacific Region, apparently following a temporal latitudinal gradient. In Karimunjawa, Indonesia (5°S), annual light and sea surface temperature variations (26–31 °C) are low, and the major spawning peak for the species complex is known to occur from March to April, with a second minor spawning peak in November [25]. The southern and eastern sides of Taiwan (21°N—25°N) are continually influenced by the warm Kuroshio Current, with sea water temperatures ranging from 23 to 29 °C year-round and coral spawning events occurring from April to May [26]. The islands west off Taiwan Island (Penghu Islands at 23°N) present annual sea water temperatures ranging from 18 to 30°C, where *A*. *hyacinthus* is known to spawn in April and May [27]. In Kochi, Japan (33°N), the annual sea water temperatures vary greatly (14–30 °C) and spawning events for *A*. *hyacinthus* have been reported to occur only during the warm months of July and August [28].

Considering the need for further research into the spatial variations in the fecundity and total reproductive output of corals [29], this study focuses on the spatial variability in the genetics and reproductive traits (i.e., gonad size and number) of the *A*. *hyacinthus* complex among a wide range of latitudinal locations (5°S to 33°N), with varying mean annual sea water temperatures (~29 °C to ~21 °C) and Photosynthetic Available Radiation (PAR) (~42 to ~32 einsteins m^-2^ day^-1^) in the Western Pacific Region. The specific questions addressed are: 1. What is the spatial variation in egg and testis size and number in *Acropora hyacinthus* at different latitudes? 2. Is the spatial variability among locations due to genetic divergence or differences in local environmental factors?

## Materials and methods

### Determining the ‘peak maturity month’ at each location

Based on previous spawning records at each location [25–28, 30]—and considering that *Acropora hyacinthus* is reportedly a split spawner [4, 31], with some exceptions reported in Japan [30]—fertility checks of 30–40 random colonies per population started one month before the reported ‘peak maturity month’ at each location, and were conducted for at least two consecutive years before and during sample collection (2012–2015). Colony fertility was checked by taking two branches of ~5 cm from at least three distinct locations within the colony (i.e., outer, mid-section and center of the colony). The species’ annual ‘peak maturity month’ per location was defined only when more than 30 colonies per location showed evidence of mature gonads (i.e., red or pink) in most colony sections, and when no gravid colonies were found the following month in the same population.

### Data collection

**Fig 1:**
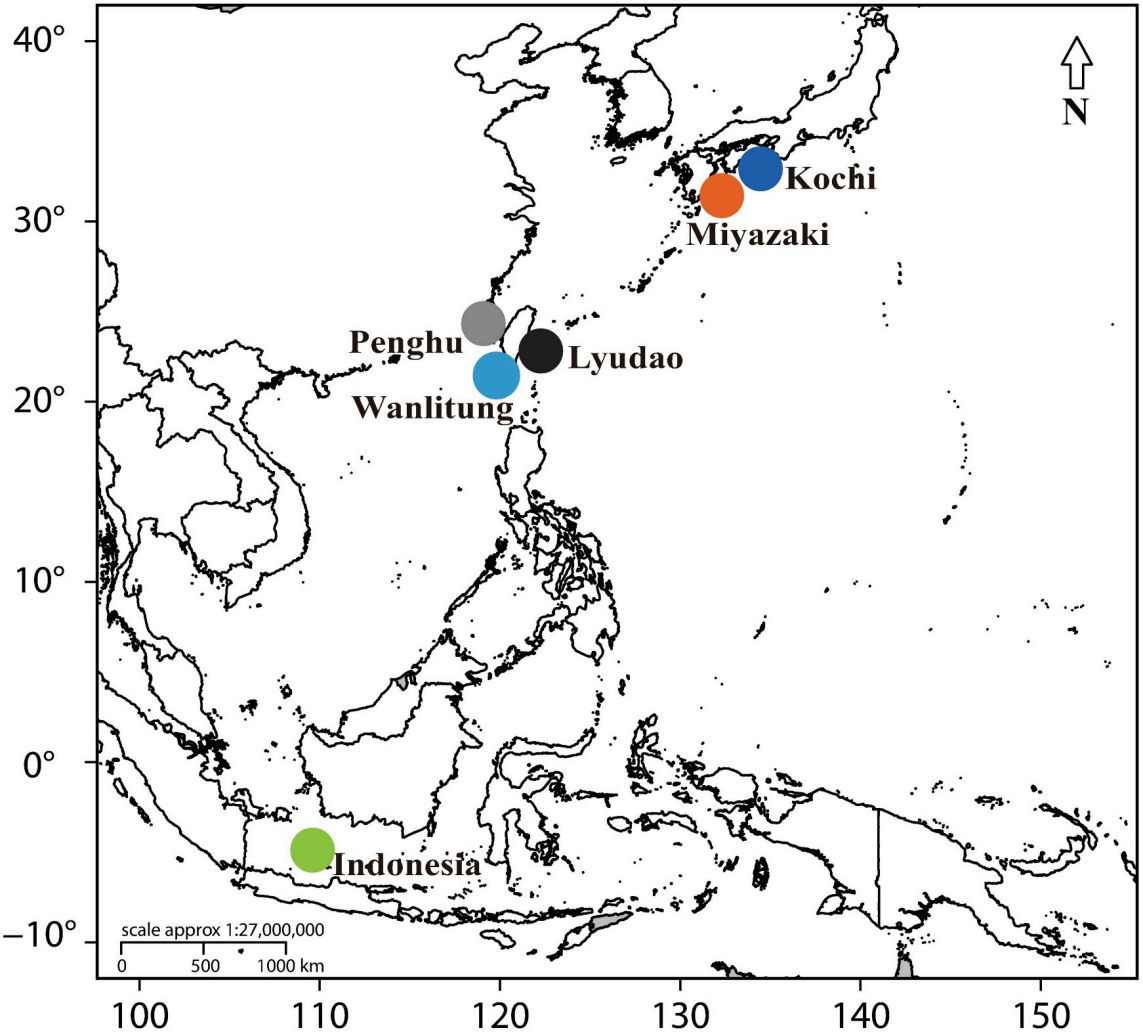
Sampled locations in the Western Pacific Region. The locations of the sampled hermaphroditic broadcast spawner *Acropora hyacinthus* in the Western Pacific Region.

Sampling was performed 1–2 weeks before the predicted spawning period of *A*. *hyacinthus* at each of the six locations in the Western Pacific Region (Fig 1), as defined by the previous fertility checks to determine the peak maturity month (pers. obsv.) and by previous published data for this species [25, 28, 30]. Due to sample permit limitations at some locations, three sites (Kochi, Japan; Lyudao, off East Taiwan; Karimunjawa, Indonesia), were sampled in 2014 and five sites (Miyazaki and Kochi, Japan; Penghu, Wanlitung, and Lyudao, Taiwan), were sampled in 2015 (S1 Table). Sampling permits at Kochi, Karimunjawa, Lyudao and Wanlitung were issued by the National Park Services at these localities, while no sampling permit was required at Miyazaki and Penghu as these were not protected areas.

Some fecundity variables of *A*. *hyacinthus* are influenced by colony size [12], so the sampled colonies consisted of medium to large size classes (168–14243 cm^2^) from shallow areas (3–5 m depth) and were separated by at least 3 m to avoid sampling clonal colonies. Considering colony inter-polyp variations, all samples were collected from the part of the colony with the highest fecundity (i.e., midway between the center and outer edges) [9]. A branch of ~5 cm in length colony^-1^ with mature gametes, judged by the pink to wine-red color of mature oocytes [32], was collected using a hammer and chisel. Sampled colonies were photographed from above using a digital camera (Canon PowerShot G16, Tokyo-Japan) to define colony size. The projected area of the colony was measured from the photographs using Adobe Photoshop CS4 software, and it is used as an index of colony size in the analyses [9]. Tissues for reproductive analyses were fixed in a 10% formalin and seawater solution. A small tissue fragment (~0.5 cm in diameter) was stored in approximately twice its volume of CHAOS-DNA digestive solution (4 M guanidine thiocyanate, 0.1% N-lauroyl sarcosin sodium, 10 mM Tris pH8, 0.1 M 2-mercaptoethanol) at room temperature [33].

Branch samples were decalcified using a solution of 10% formic acid, washed with filtered water and stored in 70% ethanol. From each sample, 15 mature polyps were randomly selected from the base of the branch and dissected by isolating all gonads under a stereoscope. Photo images of polyps and gonads were taken using the ImageJ software [34] for further measurements. Each polyp, egg and testis were measured (length and width) and counted. Estimated volumes were calculated assuming an elliptical prolate (elongated) sphere equation V = $\frac{4}{3}\pi$*ab*^2^, where ***a*** is ½ x length and ***b*** is ½ x width [12]. Because similar results were found within samples, reproductive data of five polyps per colony, in a total of 15 colonies were selected at most locations, except in Indonesia, where 12 colonies were sampled. The reproductive traits of polyp fecundity and egg size (μm means, ±SD) from all locations have been submitted to the Coral Trait Database.

During the year prior to sampling at each location, seawater temperatures were recorded at 1 hour intervals. In Indonesia and Taiwan, HOBO Pendant Data Loggers 64K (Onset, accuracy ±0.5 °C, resolution 0.14 °C) were used. In Japan, HOBO Water Temperature Pro v2 Data Loggers (Onset, accuracy ±0.2 °C, resolution 0.02 °C) provided the data. Additionally, the monthly composite values of the Photosynthetic Available Radiation (PAR) for the entire year prior to sampling were obtained from ERDDAP (http://coastwatch.pfeg.noaa.gov).

### Genetic analyses

Samples were processed following a method for DNA extractions modified from the DNeasy Blood and Tissue Kit (Qiagen, Inc., Valencia, CA, USA) manufacturer’s protocol. Part of the mitochondrial putative control region was PCR-amplified using the forward and reverse primers rns_2F: CAGAGTAAGTCGTAACATAG and block G_R: AATTCCGGTGTGTGTTCTCT, which were used in a previous study for *A*. *hyacinthus* [21]. The basic protocol for DNA amplification was 94 °C for 30 s, followed by 30 cycles of 94 °C for 20 s, 60 °C for 30 s, and 72 °C for 90 s, with a final phase of 72 °C for 5 min. Post PCR sequencing was performed by Genomics-Taipei using ABI-3730xl.

Using MEGA 7 [35], the following sequences were aligned with MUSCLE and used to construct a Maximum Likelihood (ML) tree: all sequences from the present study, the sequence region from the *A*. *hyacinthus* complete mitochondrial genome from Taiwan (accession number KF448531) and the sequence of the distantly related coral *Isopora cuneata* (outgroup) mitochondrial control region (small subunit ribosomal RNA accession number AY026429), found in the National Center for Biotechnology Information (NCBI). The H-K-Y model was selected based on the best DNA model substitution test in MEGA. Partial deletion and 1000 bootstrap replicates were selected. All positions with less than 95% site coverage were eliminated. Pairwise genetic distances among and within locations are reported (S2 Table). All DNA sequences analyzed here were submitted to the DDBJ database (accession numbers LC306704—LC306784).

Additionally, four sequences per cryptic lineage (Hya A, Hya B, HyaC and Hya D) from the different clades in the [21] study were downloaded as a blastn database from NCBI. Sequences from each phylogenetic group from the present study were used to query the blastn, and the hits of the maximum identity score and length are reported. A new phylogeny combining the sequences from both studies is also reported.

Furthermore, the entire sequence dataset was arranged using DNASP v.5 [36], and a haplotype network was developed using Pop.ART (http://popart.otago.ac.nz). For regional comparisons, haplotype groups for each location are presented in a haplotype distribution map.

### Statistical analyses

The means (±SD) and ranges (minimum-maximum) of annual seawater temperature, Photosynthetic Available Radiation (PAR), and raw data of all measured reproductive traits were calculated for each location. Two years of data are included for Kochi and Lyudao; the other locations include one year of data (n = 117).

For the overall statistical analyses, the median values of reproductive traits [egg number, median egg volume, total egg volume (sum of all eggs polyp^-1^), total testis volume (sum of all testes polyp^-1^), total gonad volume (sum of total egg volume and total testis volume)] from five polyps per colony [12] were averaged and used as a proxy for colony values [9] at each location. All data were checked for normality (Histograms and Q-Q plots) and standardized to meet the assumptions for homogeneity of variances using the statistics software R v. 3.1.1 [37]. The regional reproductive trait variability was initially assessed by a 2-dimensional Principal Component Analysis (PCA) ordination map of the combined reproductive traits for all years and locations using the ggbiplot function. Differences in environmental variables and reproductive traits among locations were evaluated using one-way ANOVA tests in conjunction with Tukey Honestly Significance Difference (HSD) multiple comparisons of means tests with significant values of p < 0.001 (Kochi and Lyudao include two years of data, other locations include one year of data). Coefficient of variation percentage values were calculated, including one year of data per location (n = 81), using the mean and standard deviation for each reproductive trait [38]. A Spearman’s rank correlation coefficient was used to assess the relationships (trade-off) between egg numbers and separate female and male gonad volumes across locations using one year of data per location, as all variables met the test assumptions (continuous, monotonic variables) [39]. In the case of median egg numbers, standardization was performed by expressing median egg numbers in relation to polyp volume (egg number per mm^3^).

Relationships between response variables (egg number per mm^3^, egg volume, total egg and testis volume and total gonad volume) and explanatory variables (numerical haplotype codes, temperature, colony area and PAR) were evaluated with a multivariate linear mixed-effect model (LMMe) using the AICcmodavg, car, lme4, MASS and MuMIn R packages [37]. Biological and physical data from the collection year were included per location (n = 81). To assess linearity and collinearity among explanatory variables, pairwise scatterplots, correlation coefficients and Variance Inflation Factor (VIF) analyses were performed with a cut-off VIF value of 3 [39]. As no collinearity was found, all explanatory variables were included in all models and dropped when found to be non-significant (p > 0.05). Explanatory variables were standardized with the R function ‘poly’. ‘Location’ was used as the random effect in all models. Models that fit the restricted maximum likelihood (REML) were considered significant for fixed effect correlation analysis. Final models were selected using the lowest Akaike Information Criterion (AIC) value. Normality of the residuals was checked using Shapiro-Wilk tests at p > 0.05 and the bell curve in a histogram [39]. Aiming to summarize the amount of variance explained by the fixed and the random factors in the models, coefficients of determination marginal and conditional R^2^ were calculated using the R package r.squaredGLMM. Marginal R^2^ (R^2^m) is concerned with the variance explained by the fixed factors (explanatory variables), and conditional R^2^ (R^2^c) is concerned with the variance explained by both fixed and random factors (entire model) [40].

## Results

**Fig 2:**
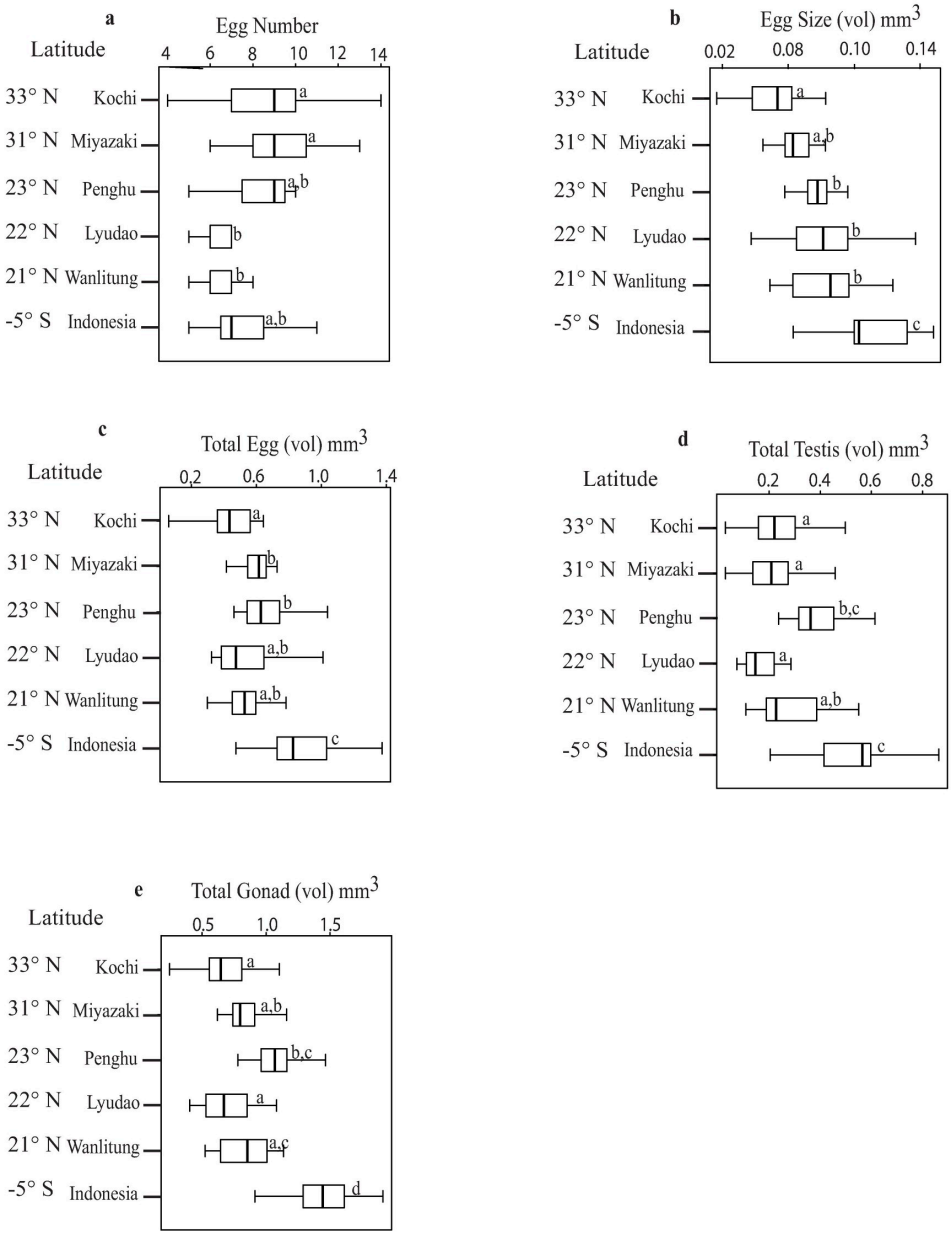
Regional variability in the reproductive traits in *Acropora hyacinthus* from the Western Pacific Region. Box plots represent latitudinal variations starting at the top with Kochi (33°N) and ending at the bottom with Indonesia (5°S). *Box plots* show all percentiles. The median (50^th^ percentile) is highlighted with a *thick bar*. Different *lowercase letters* (*a*, *b*, *c*, *d*) indicate significant results of Tukey HSD tests among locations, with values at p < 0.001. Kochi and Lyudao include two years of data; other locations include one year of data.

Significant differences in the reproductive traits of *A*.*hyacinthus* were observed among locations in the Western Pacific Region (one-way ANOVA tests, p < 0.05; Tukey HSD tests, p < 0.001; Fig 2; Table 1; S3 Table). The 2-dimensional Principal Component Analysis (PCA) of the combined traits explained 71% of the total variation. The first component PC1 (49%) presented high- and low-latitude locations distributed along the PC axis in a scattered fashion. Penghu had the least within-location variation (on PC1) among items clustered closer to each other (S1 Fig).

**Table 1:** Means and ranges of all measured reproductive and environmental variables. Summary of the means (±SD) and ranges (minimum-maximum) of annual mean sea water temperature and Photosynthetic Available Radiation (PAR), as well as the raw data of all measured reproductive traits of *Acropora hyacinthus* per location. Number of colonies per location is included (n). Lyudao and Kochi include two years of data; other locations include one year of data. Largest and lowest mean values are in *boldface*.

Variables	Kochi	Miyazaki	Penghu	Lyudao	Wanlitung	Indonesia
N	30	15	15	30	15	12
Temperature °C	**21.30 ± 3.66** **(14.71–29.72)**	23.11 ± 3.12 (16.72–30.52)	24.41 ± 2.80 (18.30–29.71)	26.72 ± 2.40 (20–31.37)	26.60 ± 2.83 (20.10–31.88)	**29.51 ± 0.82** **(26.13–31.55)**
PAR einsteins m^-2^ day^-1^	36.12 ± 11.47 (18.91–52.60)	**32.32 ± 8.72** **(21.33–47.12)**	38.72 ± 9.87 (21.91–53.43)	37.71 ± 13.30 (14.71–58.14)	42.40 ± 8.32 (23.42–54.35)	**42.53 ± 6.57** **(34.12**–**54.45)**
Egg number	8.81 ± 3.30 (0–19)	**9.48 ± 2.28** **(4–17)**	8.25 ± 2.18 (2–13)	6.78 ± 2.41 (3–14)	**6.42 ± 1.61** **(4–11)**	7.55 ± 2.07 (4–13)
Egg size volume mm^3^	**0.05 ± 0.01** **(0–0.10)**	0.06 ± 0.01 (0.04–0.12)	0.08 ± 0.01 (0.04–0.16)	0.08 ± 0.03 (0.03–0.19)	0.09 ± 0.03 (0.03–0.23)	**0.11 ± 0.02** **(0.06–0.17)**
Total egg volume mm^3^	**0.44 ± 0.19** **(0–1.35)**	0.63 ± 0.19 (0.25–1.57)	0.66 ± 0.20 (0.21–1.26)	0.58 ± 0.30 (0.21–1.72)	0.53 ± 0.17 (0.21–0.95)	**0.88 ± 0.34** **(0.29–1.82)**
Total testis volume mm^3^	0.24 ± 0.13 (0.02–0.63)	0.21 ± 0.13 (0–0.59)	0.39 ± 0.17 (0.10–1.01)	**0.18 ± 0.13** **(0.04–0.97)**	0.29 ± 0.15 (0.04–0.67)	**0.56 ± 0.28** **(0.12–1.61)**
Total gonad volume mm^3^	**0.69 ± 0.24** **(0.15–1.68)**	0.84 ± 0.24 (0.28–1.77)	1.05 ± 0.29 (0.49–1.69)	0.77 ± 0.41 (0.27–2.44)	0.83 ± 0.27 (0.31–1.44)	**1.44 ± 0.52** **(0.50–2.96)**

The results from the multiple comparisons of means tests indicated significant differences in egg numbers only among the two high-latitude (Kochi and Miyazaki) and two mid-latitude (Lyudao and Wanlitung) locations (Tukey HSD test, p < 0.001; Fig 2a). Samples from one high-latitude location presented the highest egg number coefficient of variation (Kochi, 33°N; CV = 34%), while the lowest was found at the lowest latitude (Indonesia, 5°S; CV = 24%), among all locations (Table 2).

**Table 2:** Annual variations in the reproductive traits of *Acropora hyacinthus* across locations.

Latitude Reproductive traits	Kochi 33°N	Miyazaki 31°N	Penghu 23°N	Lyudao 22°N	Wanlitung 21°N	Indonesia 5°S
*Egg number per mm^3^*	**34**	25	24	29	26	**23**
*Egg volume*	35	16	**13**	**46**	37	16
*Total egg volume*	32	**24**	**24**	**63**	27	32
*Total testis volume*	52	57	**28**	**86**	46	31
*Total gonad volume*	27	**21**	23	**66**	25	25

The Coefficient of Variance (CV%) of all measured reproductive traits per location was calculated using the mean and standard deviation value. Biological data of the collection year were included per location (2014 = Indonesia; 2015 = Kochi, Miyazaki, Penghu, Lyudao and Wanlitung). Highest and lowest values in *boldface*.

Significant differences in egg volume among high-, mid- and low-latitude locations are reported (Tukey HSD test, p < 0.001; Fig 2b). The largest mean egg volume values were observed in Indonesia and the smallest in Kochi and Miyazaki (Table 1). Samples from two mid-latitude locations had the highest coefficient of variation (Lyudao, 22°N; CV = 46%) and the lowest (Penghu, 23°N; CV = 13%) for egg volume among all locations (Table 2).

The total egg volume significantly varied among both high- and mid-latitude locations and the lowest latitude location (Tukey HSD test, p < 0.001, Fig 2c). Indonesia (the lowest latitude location, 5°S) had the largest total egg volume mean value and Kochi (a high-latitude location, 33°N) had the smallest (Table 1), although the latter location was not significantly different in total egg volume when compared to Lyudao and Wanlitung (two mid-latitude locations, 22°N and 21°N, respectively). One mid-latitude location had the highest total egg volume coefficient of variance (Lyudao, 22°N; CV = 63%), whereas one mid-latitude (Penghu, 23°N) and one high-latitude location (Miyazaki, 31°N) shared the lowest values, respectively (CV = 24%; Table 2).

There were no significant differences in total testis volume among high-latitude and two mid-latitude locations. However, a mid-latitude location (Penghu, 23°N), was significantly different from the two high-latitude (Kochi, 33°N and Miyazaki, 31°N) and one mid-latitude (Lyudao, 22°N) locations, but the total testis volume at this location was similar to other mid-latitude location (Wanlitung, 21°N) and to the lowest latitude location (Indonesia, 5°S) (Tukey HSD test, p < 0.001, Fig 2d). The largest mean total testis volume value was observed for Indonesia and the smallest for Lyudao (Table 1). Two mid-latitude locations had both the highest total testis volume coefficient of variance (Lyudao, 22°N; CV = 86%) and the lowest (Penghu, 23°N; CV = 28%) among all locations (Table 2).

Finally, total gonad volumes varied significantly between Indonesia and all other latitude locations (Tukey HSD test, p < 0.001, Fig 2e). Penghu was statistically different from Lyudao (another mid-latitude location, 22°N) and to Kochi (a high-latitude location, 33°N), but not to Wanlitung (a mid-latitude location, 21°N) and Miyazaki (a high-latitude location, 31°N). The highest mean total gonad volume value was found in Indonesia, whereas the smallest value was found in Kochi (Table 1). Lyudao (a mid-latitude location, 22°N) had the highest total gonad volume coefficient of variance (CV = 66%), while Miyazaki (a high-latitude location, 31°N) had the lowest (CV = 21%) among all locations (Table 2).

**Fig 3:**
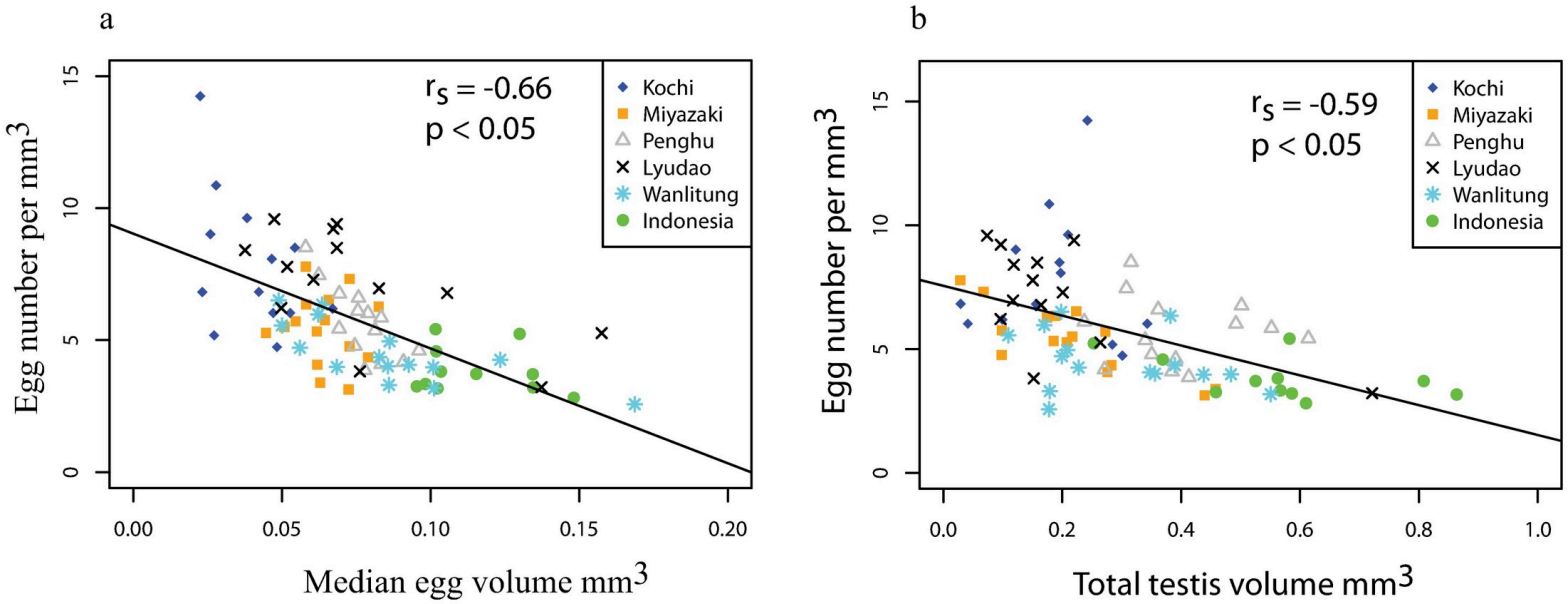
Reproductive trade-offs in *Acropora hyacinthus* from the Western Pacific Region. **a.** The relations of egg numbers per mm^3^ vs median egg volume (mm^3^) **b.** The relations between egg numbers per mm^3^ vs total testis volume (mm^3^). The results of the Spearman’s rank correlation coefficient are reported. One year of data (n = 81) is included per location (2014 = Indonesia; 2015 = Kochi, Miyazaki, Penghu, Lyudao and Wanlitung).

There was a significant negative correlation between egg number per mm^3^ and both median egg volume (Spearman correlation coefficient, r_s_ = -0.6675, p < 0.05; Fig 3a) and total testis volume (Spearman correlation coefficient, r_s_ = -0.5985, p < 0.05; Fig 3b), indicating reproductive trade-offs.

### Relations between reproductive traits and haplotype, temperature, colony area and PAR

The results from the linear mixed models indicated that haplotype had a significant relation with both egg number per mm^3^ (square root transformed LMMe, p < 0.05) and egg volume (log-transformed LMMe, p < 0.05); however, in both models the haplotype effect accounted for less than 1% of the variation (R^2^m). In the case of the fecundity model (egg number per mm^3^), R^2^m (all fixed factors included) explained 23% of the variation in the model, and this value increased to 45% when the random factor of ‘location’ was included (R^2^c). On the other hand, in the egg volume model, R^2^m values (haplotype and annual mean temperature together) explained up to 52% of the variance, with a small increase to 54% when the random factor (R^2^c) was included. This indicates a very large effect by the fixed factors (i.e., Haplotype = 3%, Temperature = 49%) in the egg volume model, and agrees with the significantly positive relation between annual mean seawater temperature and both individual egg volume and total egg volume (log-transformed LMMe, p < 0.001). However, only 19% of the variation is explained by annual mean temperature (R^2^m) in the total egg volume model, while a larger effect is observed when the random factor of location is added (R^2^c = 41%).

Lastly, there was a significant positive relation between total testis volume and colony area (square root transformed LMMe, p < 0.05). However, in this model, the fixed factor effect of colony area accounted for only 5% of the variance in the total testis model, while a very large effect was observed when the random factor of location was added (R^2^c = 57%). PAR had no significant relationship with any one reproductive trait (S4 Table).

### Genetic analyses

**Fig 4:**
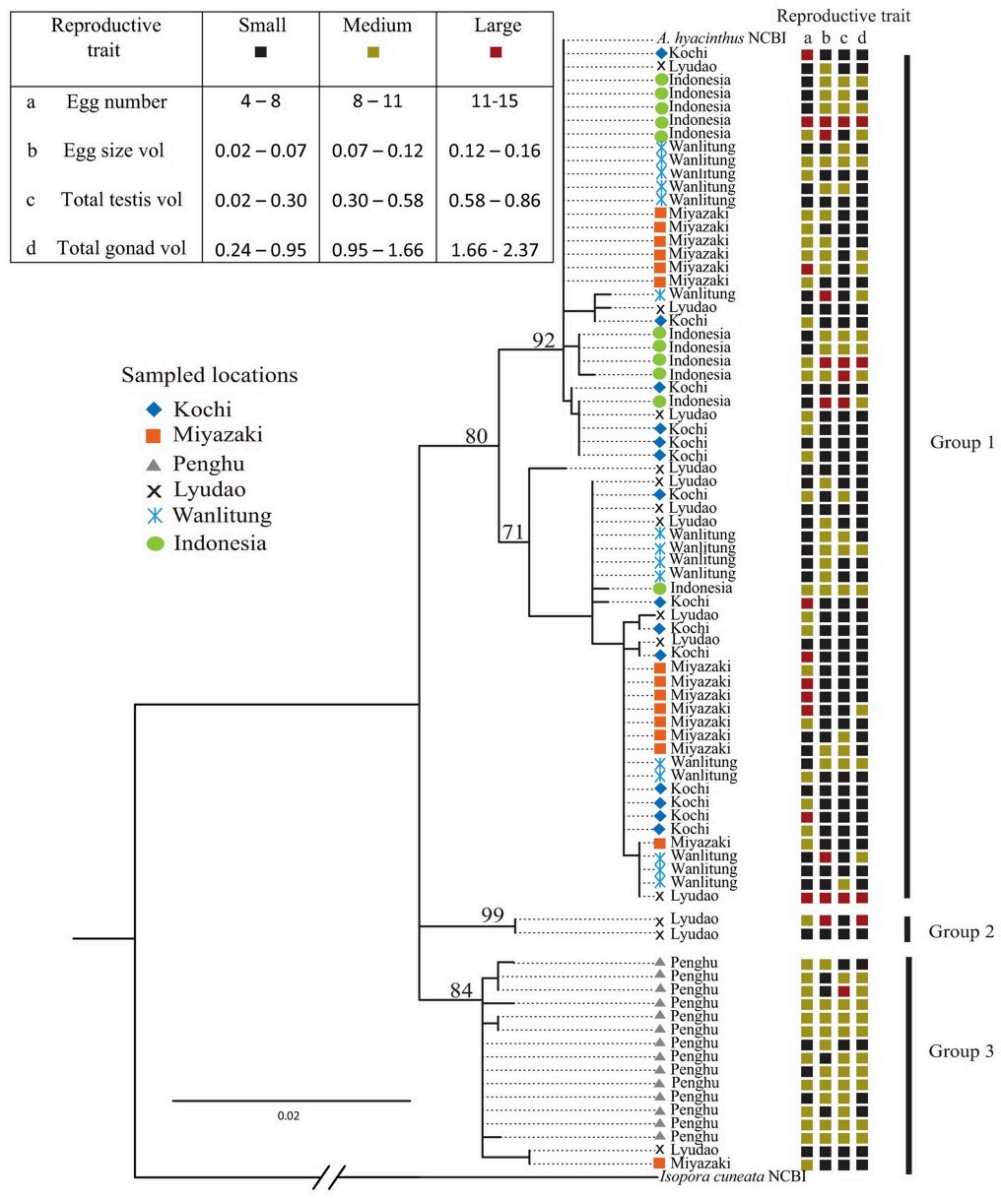
Genetic and reproductive traits in *Acropora hyacinthus* from the Western Pacific Region. The evolutionary history was inferred based on 767 bp of the putative mitochondrial control region (mtCR) of *Acropora hyacinthus* using the maximum likelihood method based on the H-K-Y model and 1000 bootstrap replications. The *bootstrap support value* is shown next to the branches, and 3 groups were found. *Acropora hyacinthus* (acc. Number KF448531) was included, and *Isopora cuneata* (acc. number AY026429) was used as an outgroup. Next to each sample location, *colored squares* indicate the calculated *size class*—*small (black)*, *medium (green) and large (red)*—for the locations of main reproductive traits using the formula: $\frac{\text{max}\;\text{measurement}\;-\text{min}\;measurement+1}{size\;class\;categories\;(3)}$.

The sampled *A*. *hyacinthus* were classified into three distinct groups (bootstraps > 80) in the maximum likelihood (ML) tree (Fig 4). Group One was a mixture of samples from all locations, except Penghu. Group Two included two samples from Lyudao, and Group Three included all samples from Penghu and one sample each from Lyudao and Miyazaki. Additionally, the sequences from all three groups in the present study showed similarities to the cryptic lineages Hya A, B, C and D previously reported for the Kuroshio Region [21] (S2 Fig and S5 Table).

**Fig 5:**
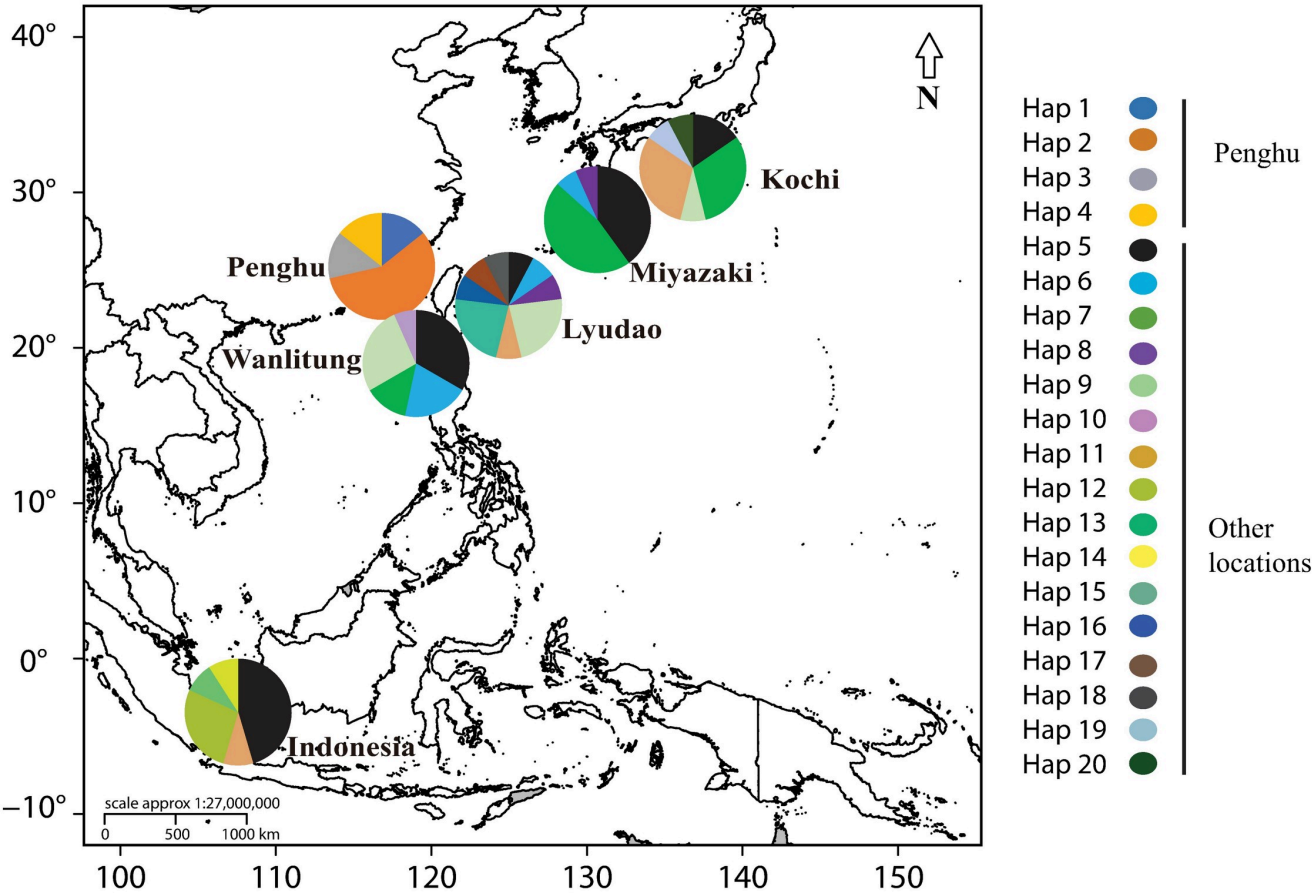
Haplotype distribution of the *Acropora hyacinthus* species complex in the Western Pacific Region. The distribution of the 20 haplotypes found at the six sampled locations.

A total of 20 haplotypes were found in mixed fashion at all locations except Penghu, where four unique haplotypes were identified (Fig 5). The other 16 haplotypes were scattered among Indonesia (5 haplotypes), Wanlitung (5 haplotypes), Lyudao (9 haplotypes), Miyazaki (4 haplotypes) and Kochi (6 haplotypes). Only one haplotype (Hap 5) was found in common at all of these five locations. High genetic distances were found among haplotypes, even from the same location (S3 Fig).

## Discussion

This study reports the spatial variability in the reproductive traits of *Acropora hyacinthus* among six different latitudinal locations in the Western Pacific Region. Egg volumes were larger at the lowest latitude location—significantly decreasing as the location latitude increased—and were significantly positively correlated with annual mean seawater temperature. Consequently, egg number was significantly negatively correlated with both gonad volumes across locations, indicating reproductive trade-offs. These results suggest that the gametic biology dynamics of *A*. *hyacinthus* are strongly influenced by some selective pressure(s) that varies with latitude, such as sea water temperature. To my knowledge, this is the first report of a coral species producing smaller egg sizes at higher latitudes and larger egg sizes as latitude decreases.

It is not immediately clear from these results why the coral produces significantly smaller egg sizes at the highest latitude, medium sizes at mid-latitudes and larger sizes at the lowest latitude location. However, since both gonad volumes decrease with latitude (linear regression p < 0.001; S5 Fig), the non-significant difference in egg numbers at high and low latitudes cannot be explained by latitudinal variations in the total energetic investment in eggs and testes, but rather by a trade-off between gonad size and egg number, as has been reported in other aquatic animals (e.g., Fish [41]). Indeed, the significant negative correlations between egg numbers and both female and male gonad volumes is strong across locations, but no significance was found within some locations (S6 Fig). Thus, the implication of a trade-off among egg numbers and both gonad volumes across locations, as well as the overall higher contribution of the random effect (i.e., location) in most statistical models, implies differences in local parental effects (e.g., parental investment), phenotype (e.g., colony structure) and genotype (e.g., hybridization) under prevailing local environmental conditions [42].

Even though total testis volume was significantly positively correlated with colony area (S4 Table), agreeing with a previous study of the species from a high-latitude location [9], much of the variability resided in the location effect (R^2^c = 57%). Additionally, all measured reproductive traits varied greatly, even among colonies of similar sizes from the same and different latitudinal locations (S4 Fig). Hence, considering this study sampled sexually mature colonies, the differences in reproductive traits (i.e., spatial variability) among locations was not a function of different colony sizes, but possibly of non-measured site-to-site differences, as has been reported in brooding species [10]. These may or may not have involved climatic variables. Furthermore, higher variability was found in both gonad volumes at one mid-latitude location (i.e., Lyudao, Table 2, S5 Fig). Overall, this suggests that, regardless of their location, reproductive mother colonies of the broadcast spawner *A*. *hyacinthus* may maximize their own and their offspring’s fitness, considering their unpredictable future environment [38], by increasing the intra colony variation in trait phenotypes within a gametogenic cycle. Future studies, including the analyses of consecutive reproductive cycles and seasonal changes (e.g., environmental color [43]) at different locations with similar latitude, may assist in understanding the reported spatial variations.

A plausible explanation for the regional differences in both gonad volumes is that there is a negative relationship between latitude and sea water temperature, which coincides with the significant positive relationship between female gonad volumes and seawater temperature (S7 Fig). Because corals are poikilotherms, variations in the mean sea water temperature are known to affect the energy available for reproduction [44]; for example, high sea water temperatures have been correlated with a reduction in larval production in brooders such as *Acropora palifera* from a tropical location [11] and *Porites astreoides* from a high-latitude reef [45], while an increase in gonad development associated with an increase in sea water temperatures has been reported in tropical gonochoric broadcasting corals [46]. Previous studies on high latitude coral reproduction have suggested that lower mean seawater temperatures delay the rate of coral gamete maturation (e.g., *Alveopora japonica* [47]), a characteristic that has also been found in soft corals [48]. This may partially explain why the single annual gametogenic cycle of *A*. *hyacinthus* was reported at higher latitudes (e.g., Japan [28]), while in Indonesia it occurs twice per year [25], but it does not explain the significant correlation between the production of larger female gonad volumes at tropical locations and sea water temperature, as reported in the present study. A comparative study in gametogenic cycle lengths and metabolic costs of reproduction at different latitudes is still needed.

While the interactions reported in this study do not necessarily prove causality, seawater temperature has a well-documented negative correlation with egg size. Marine animals that live at low temperatures often produce larger eggs compared to their tropical congeners [13, 14, 49], and those exposed to high temperatures produce smaller eggs (e.g., *Dinophilus gyrociliatus* [50]). Nevertheless, there are cases of positive correlations between egg size and temperature in octocorals [15, 48], and these correlations have been attributed to differences in the energy available to the mother colony during gametogenesis.

Annual mean PAR did not correlate with any one trait, but the potential light effect (e.g., environmental color [51]) on the total energetic proportion invested by mother colonies throughout the gametogenetic cycle at a particular location [52] cannot be discarded. Considering that tropical waters are known to be less productive [53], which may compromise post-spawning development, eggs produced by colonies exposed to higher light conditions may have increased amounts of biochemical components—such as wax esters [54]—resulting in larger gonad sizes that may favor adaptations for better post-settlement performance in *A*. *hyacinthus* larvae. Likewise, factors such as heterotrophic energy acquirements [55, 56] and the type of endolithic algae at each location or in parts of the colony [57] may also reflect variations in the net values of the biochemical components that will be translated into the total energy allocated for reproduction in *A*. *hyacinthus*.

On the other hand, the reproductive benefit for the increased variance in egg numbers of small sizes at high latitudes may represent a better chance for fertilization and wide dispersal, considering extreme local conditions [58] or the higher environmental seasonality [43] at these locations. Such a strategy may favor the restriction of *A*. *hyacinthus* to marginal locations with high environmental instability, allowing it to successfully reproduce and thrive, and contributing to the maintenance and proliferation of local marginal populations [59]. Likewise, considering the unpredictable environments of *A*. *hyacinthus* offspring, the benefits of the production of large and low egg numbers and sizes produced at the lowest latitude location may correspond to higher energetic lipid reserves in the higher coral cover populations [29] of *A*. *hyacinthus* found in the tropical Indo-Pacific, reflecting strategies of increased resource allocation in increasing fecundity and diversifying phenotypes (i.e., bet-hedging strategy [38], but see [43]); this may, in turn, improve the chances for fertilization [60] and survival at a specific location [61]. Furthermore, larger eggs may carry greater nutrient stores and thus result in longer-lived larvae [62]; thus, the production of larger egg sizes in the tropics may be actively contributing to the wide distribution of the species complex at latitudinal and longitudinal dimensions in the Indo-Pacific Ocean [20]. Future latitudinal comparative studies in the larval developmental mode of the species may help explain the spatial variations reported here.

Further research monitoring sea water temperature and light irradiance at the colony depth using reciprocal transplantation experiments of mother colonies would help further analyze phenotypic plasticity in *A*. *hyacinthus*. By examining maternal age, size and the biochemical compositions of original and transplanted colonies, data on the egg volumes during sequential gametogenic cycles in cold/warm environments will assist in understanding the benefits of the observed latitudinal variations related to coral reproductive success.

Differences in the reproductive traits among locations are not because of genetic divergence. The genetic composition of *A*. *hyacinthus*, as determined using the mitochondrial control region (mtCR), was similar among low-, mid- and high-latitude locations (except for Penghu), suggesting ongoing gene flow from tropical to marginal locations. This gene flow may be due to the continual northward Kuroshio Current, which is known to act as a conveyor for larval transport from tropical to subtropical and temperate corals [63], and assists in the occurrence of the widely dispersed syngameon of *A*. *hyacinthus* morph-species in the Western Pacific Region [23].

The present study was not definitive on specific cryptic lineages at each location, but a similar colony morph structure may mask the occurrence of sympatrically distributed cryptic species within the *A*. *hyacinthus* complex. Lyudao had the highest variability among most reproductive traits and number of haplotypes; also, two samples were grouped independently from the other two groups in the present study and to previously reported sequences [21]. The possibility that these two samples came from the same individual (genets) cannot be dismissed considering the relatively high occurrence of typhoons in the area, which may allow for a high local clone dispersion. A thorough examination of these two colonies confirmed that both sequences, although short in length, presented high identity proportions with the *A*. *hyacinthus* HyaC cryptic lineage in the [21] study. They also have similar colony morphs, reproductive structures and spawning times (author pers. obsv.) in comparison to other colonies from the same area, which, grouped in a mixed fashion (Groups One and Three) in the phylogeny, suggest no reproductive isolation within colonies at this location. However, the occurrence of these two genetically unique, highly genetically variable samples alongside the physiological similarities with all of the other sampled colonies at this location, suggest sympatric subdivisions (“endemic singameons”) among cryptic *Acropora* morph-species [21, 23, 64]. Further genetic analyses in combination with reproductive and cross fertilization studies will help reject or support this hypothesis.

Penghu presented higher genetic and reproductive similarities compared to the other locations, implying reproductive isolation possibly due to vicariant allopatric subdivisions (e.g., influenced by ocean currents). *A*. *hyacinthus* from Penghu formed an independent group in the phylogeny, exhibited a low and “unique” haplotype composition, had the smallest within-location genetic distances, had the highest among-locations genetic distances (S2 Table), and presented high identity proportions with the cryptic lineage HyaC reported for this area by [21]. Additionally, a higher similarity of factors in the PCA analysis and low proportions in coefficient of variance values indicated higher similarity in reproductive traits at this location. The evidence of low genetic distances within this group, in contrast to the large genetic distances among colonies from all other locations, suggest restricted gene flow between this and the other sampled locations, possibly indicating a greater potential for endemic syngameon lineage/s of sympatric cryptic and/or sister species within *A*. *hyacinthus* [23].

The differences mentioned in *A*. *hyacinthus* from Penghu and the other locations may be explained by historical events, such as exposure to low winter seawater temperatures in the Penghu Islands due to the cold (~16 °C) Southward China Coastal Current [65], which may seasonally diminish the intensity of the warm Kuroshio Current that continually runs northward along the east coast of Taiwan, limiting gene flow between the eastern and western sides of the island. Additionally, there is geologic evidence that a residual land bridge connected Taiwan to mainland China until ~7500 years ago [66], which may have acted as a partial barrier for gene flow between this location and the other sampled locations. The reproductive and genetic similarities within Penghu colonies, accompanied by the high genetic distance, different colony morphologies [24] and spawning time with all other locations, suggest the potential for local adaptations of *A*. *hyacinthus* to the distinctive local environmental conditions of Penghu Islands, further supporting the concept of reproductive isolation in the *A*. *hyacinthus* species complex in Penghu previously reported by [27].

## Conclusions

Overall, this study reports spatial variations in the reproductive traits of gonad size and number in *A*. *hyacinthus* in the Western Pacific Region. The maximum egg numbers and minimum egg sizes were observed at high latitudes, which also presented the highest variability in egg numbers (i.e., Kochi). However, there were no significant differences in egg number between the two high-latitude locations, one of the mid-latitude locations (Penghu), and the lowest latitude location. Eggs were significantly smaller in number between two mid-latitude locations (i.e., Lyudao and Wanlitung) and the two high-latitude locations.

The lowest latitude location (Indonesia) had significantly larger egg sizes, total egg and total gonad volumes compared to all other locations. Indonesia also presented the largest total testis volume among all locations, but the mean volume values were not significantly different to two mid-latitude locations (Penghu and Wanlitung). Although the causes for the observed regional variations have not been defined, considering the active gene flow reported from low- to high-latitude locations, natural selection is thought to modify those life history traits, which may provide a fitness advantage under local environmental conditions [67, 68]. Hence, the reported significant positive interactions between female gonad volumes and seawater temperature, the significant negative relations among egg numbers and both gonad volumes across locations indicating reproductive trade-offs, as well as the large effects of the random factor of ‘location’ in all statistical models, suggest that the combination of colony energetics and environmental conditions at a local level are likely to contribute to the observed spatial variability. Finally, the combination of genetic and reproductive similarities implies that the isolated population of *A*. *hyacinthus* from Penghu has the potential for local adaptations to the conditions (e.g., low winter sea water temperatures) of this location. Since the species complex is widely distributed from tropical to high-latitude locations in the Indo-Pacific Region, the reported regional variations and local similarities in the reproductive and genetic traits, suggest differential advantages and adaptive potentials within the species complex. Furthermore, and especially in consideration of current, rapid climate change scenarios, *A*. *hyacinthus’* apparent adaptive potentials make it an important species candidate in coral reef restoration practices, and as such demand our heightened attention.

## Supporting information

S1 Fig The 2 dimensional PCA ordination of the combined reproductive traits. Kochi and Lyudao includes two years of data, other sites include one year of data. Traits include: egg number, median values of eggs, total testis volume and total gonad volume.
(PDF)

S2 Fig Comparative genetic analysis with a previous study in the penghu area. The evolutionary history was inferred based on 767 bp of the putative mitochondrial control region (mtCR) of *Acropora hyacinthus* from the present study and at least two sequences from each of the reported cryptic lineages sequences (Hya A, B, C and D) from each clade in the phylogeny tree from Suzuki et al (2016), stored in NCBI. The previously used *Acropora hyacinthus* sequence from NCBI (acc. Number KF448531) was included and *Isopora cuneata* (acc. number AY026429) was used as outgroup. The maximum likelihood method based on the H-K-Y model and 1000 bootstrap was used. The bootstrap support group is shown next to the branches.
(PDF)

S3 Fig *Acropora hyacinthus* haplotype network in the Western Pacific Region. Colors indicate the sampled location. The size of each pie represents the number of similar haplotypes.
(PDF)

S4 Fig Reproductive traits and colony area relations. The relations between all reproductive traits and colony area at all locations using non-transformed data (n = 111). Kochi and Lyudao include two years of data. Colored icons indicate the sampled location. When significance was detected, linear regression results of p, F and R^2^ values are included.
(PDF)

S5 Fig Latitudinal variability in the reproductive traits in *Acropora hyacitnhus*. Results of the linear regression analyses are included. Shaded area = 95% confidence interval.
(PDF)

S6 Fig Reproductive traits correlations in *Acropora hyacinthus* at all sampled locations in the Western Pacific Region. **a.** The relations of Egg numbers per mm^3^ vs median egg volume (mm^3^) **b.** The relations between Egg numbers per mm^3^ vs total testis volume (mm^3^). The results of the Spearman’s rank correlation coefficient are reported. One year of data is included per location [2014 = Indonesia (n = 12); 2015 = Kochi, Miyazaki, Penghu, Lyudao and Wanlitung (n = 15 per location)].
(PDF)

S7 Fig Reproductive traits and annual mean sea water temperature relations. The relations between all reproductive traits and sea water temperature at all locations using non-transformed data (n = 81). Biological and physical data of the collection year were included per location (2014 = Indonesia; 2015 = Kochi, Miyazaki, Penghu, Lyudao and Wanlitung). Colored icons indicate the sampled location. When significance was detected, linear regression results of p, F and R^2^ values are included.
(PDF)

S8 Fig Pairwise scatterplots with correlation coefficients. (PDF)

S9 Fig R scripts of the linear mixed models. (R)

S1 Table The six sampled locations in the Western Pacific Region. Relevant information includes latitude, longitude and sample month(s) per year(s) at each location.
(PDF)

S2 Table Genetic distance within and among locations. Highest genetic distance values among locations are in *boldface*.
(PDF)

S3 Table Significant regional differences among the reproductive traits in *Acropora hyacinthus*. Summary of the one-way ANOVA results of the annual mean temperature and PAR and all measured reproductive traits across locations (Kochi and Lyudao include two years of data; the other locations include one year of data. Degree of freedom = 75. Significant p values in *boldface*.
(PDF)

S4 Table Relationships between response and explanatory variables using LMMe. The relationship between response variables (reproductive traits) and explanatory variables [Annual mean Temperature and Photosynthetic Available Radiation (PAR), colony area and haplotype] using linear mixed models. ‘Location’ was included as the random variable. Summary of ANOVA tests with significant values at p < 0.05 in *boldface*. Biological and physical data of the collection year were included per location (2014 = Indonesia; 2015 = Kochi, Miyazaki, Penghu, Lyudao and Wanlitung).
(PDF)

S5 Table Haplotype similarities to previous reports. Results of the blastn analysis using four sequences per lineage (HyaA, HyaB, HyaC and HyaD) from the different clades in the Suzuki et al. (2016) study downloaded as a blastn database. At least two sequences from each lineage group from the present study were used as query to perform the blastn. The maximum identity score hit from the present study sequences with the respective sequence length and those lineage ID sequences reported in the Suzuki et al. (2016) study are included in *red*.
(PDF)

S6 Table Biological and physical measurements from all locations studied. Data submitted to the Coral Trait Database (egg number and size μm means, ±SD) and additional information of all response and explanatory variables (medians and coefficient of variance).
(XLSX)

## References

[1] Brown C, Laland KN. Social learning in fishes: A review. Fish and Fisheries 2003;4:280–8.

[2] Kasyanov VL. Reproductive strategy of marine bivalves and echinoderms: Oxonian Press, New Delhi; 2001.

[3] Laptikhovsky V. Latitudinal and bathymetric trends in egg size variation: a new look at Thorson’s and Rass’s rules. Mar Ecol Prog Ser. 2006;27(1):7–14.

[4] Baird AH, Guest JR, Willis BL. Systematic and biogeographical patterns in the reproductive biology of scleractinian corals. Annu Rev Ecol Evol Syst. 2009;40(1):551–71.

[5] Keith SA, Maynard JA, Edwards AJ, Guest JR, Bauman AG, van Hooidonk R, et al Coral mass spawning predicted by rapid seasonal rise in ocean temperature. Proc Biol Sci. 2016;283(1830). 10.1098/rspb.2016.0011 27170709 PMC4874704

[6] Richmond RH, Hunter CL. Reproduction and recruitment of corals: comparisons among the Caribbean, the tropical Pacific, and the Red Sea. Mar Ecol Prog Ser. 1990;60:185–203.

[7] Wallace CC. Reproduction, recruitment and fragmentation in nine sympatric species of the coral genus *Acropora*. Marine Biology. 1985;88(3):217–33.

[8] Harrison PL, Wallace CC. Reproduction, dispersal and recruitment of scleractinian corals In: Dubinsky Z., (ed) Coral Reefs: Ecosystems of the world; Elsevier, Amsterdam, Netherlands 1990;25:133–207.

[9] Nozawa Y, Lin CH. Effects of colony size and polyp position on polyp fecundity in the scleractinian coral genus *Acropora*. Coral Reefs. 2014;33(4):1057–66.

[10] de Putron SJ, Lawson JM, White KQ, Costa MT, Geronimus MV, MacCarthy A. Variation in larval properties of the Atlantic brooding coral *Porites astreoides* between different reef sites in Bermuda. Coral Reefs. 2017;36(2):383–93.

[11] Kojis BL, Quinn NJ. Seasonal and depth variation in fecundity of *Acropora palifera* at two reefs in Papua New Guinea. Coral reefs. 1984;3(3):165–72.

[12] Hall VR, Hughes TP. Reproductive strategies of modular organisms: Comparative studies of reef building corals. Ecol 1996;77:950–63.

[13] Sier C, Olive P. Reproduction and reproductive variability in the coral *Pocillopora verrucosa* from the Republic of Maldives. Marine Biology. 1994;118(4):713–22.

[14] Fan TY, Dai CF. Reproductive plasticity in the reef coral *Echinopora lamellosa*. Mar Ecol Prog Ser. 1999;190:297–301.

[15] Tsounis G, Rossi S, Aranguren M, Gili JM, Arntz W. Effects of spatial variability and colony size on the reproductive output and gonadal development cycle of the Mediterranean red coral (*Corallium rubrum L*.*)*. Mar Biol. 2006;148(3):513–27.

[16] Dahan M, Benayahu Y. Reproduction of *Dendrodephthya Hemprichi* (Cnidaria:Octocorallia): Year-round spawning in a zooxanthellate soft coral. Marine Biology. 1997;129:573–9.

[17] Fan TY, Chou YH, Dai CF. Sexual reproduction of the Alcyonacean coral *Lobophytum pauciflorum* in southern Taiwan. Bull Mar Sci 2005;76(1):143–54.

[18] Benayahu Y, Loya Y. Sexual reproduction of a soft coral: Synchronous and brief annual spawning of *Sarcophyton glaucum* (quoy & gaimard, 1833). Biol Bull. 1986;170:32–42.

[19] Kruger A, Schleyer MH, Benayahu Y. Reproduction in *Athelia glauca* (octocorallia:Xeniidae). I. Gametogenesis and larval brooding. Mar Biol. 1998;131:423–32.

[20] Veron JE. Corals of the world, vol. 1–3. Australian Institute of Marine Science, Townsville. 2000:404–5.

[21] Suzuki G, Keshavmurthy S, Hayashibara T, Wallace CC, Shirayama Y, Chen CA, et al Genetic evidence of peripheral isolation and low diversity in marginal populations of the *Acropora hyacinthus* complex. Coral Reefs. 2016;35:1419–32.

[22] Van-Oppen MJH, Willis BL, Rheede TV, Miller DJ. Spawning times, reproductive compatibilities and genetic structuring in the *Acropora aspera* group: evidence for natural hybridization and semi-permeable species boundaries in corals. Mol Ecol 2002;11:1363–76. 12144658 10.1046/j.1365-294x.2002.01527.x

[23] Ladner JT, Palumbi SR. Extensive sympatry, cryptic diversity and introgression throughout the geographic distribution of two coral species complexes. Mol Ecol. 2012;21(9):2224–38. 10.1111/j.1365-294X.2012.05528.x . 22439812

[24] Dai CF, Horng S. Scleractinia fauna of Taiwan: Complex group: National Taiwan University Press, Taipei, Taiwan; 2009.

[25] Permata D, Indrayanti E, Haryanti D, Fika L, Arfiyan H, Achmad A. Biannual multispecific spawning in Karimunjawa Archipelago, Indonesia. Coral Reefs. 2012;31(3):907-.

[26] Dai CF, Soong K, Fan TY. Sexual reproduction of corals in northern and southern Taiwan. Proc 6^th^ Int Coral Reef Symp. 1992;I:448–55.

[27] Wei NV, Chen CA, Hsieh HJ, Dai CF, Wallace CC, Baird AH. Reproductive isolation among *Acropora* species (scleractinia: Acroporidae) in a marginal coral assemblage. Zool Stud. 2012;51(1):85–92

[28] Mezaki T, Hayashi T, Iwase F, Nakachi S, Nozawa Y, Miyamoto M, et al Spawning patterns of high latitude scleractinian corals from 2002 to 2006 at Nishidomari, Otsuki, Kochi, Japan. Kuroshio Biosphere. 2007;3:33–47.

[29] Hartmann AC, Marhaver KL, Vermeij MJ. Corals in healthy populations produce more larvae per unit cover. Conservation Letters. 2017.

[30] Nozawa Y. Annual variation in the timing of coral spawning in a high-latitude environment: Influence of temperature. Biol Bull 2012;222:192–202 10.1086/BBLv222n3p192 22815368

[31] Foster T, Heyward AJ, Gilmour JP. Split spawning realigns coral reproduction with optimal environmental windows. Nature communications. 2018;9(1):718 10.1038/s41467-018-03175-2 29459700 PMC5818648

[32] Baird AH, Marshall PA, Wolstenholme J. Latitudinal variation in the reproduction of *Acropora* in the Coral Sea. Proc 9^th^ Int Coral Reef Symp. 2000;1:23–7

[33] Fukami H, Budd AF, Levitan DR, Jara J, Kersanach R, Knowlton N. Geographic differences in species boundaries among members of the *Montastraea annularis* complex based on molecular and morphological markers. Evolution. 2004;58(2):324–37. 15068349

[34] Abramoff MD, Magalhaes PJ, Ram SJ. Image Processing with ImageJ. Biophot Intern. 2004;11:36–42.

[35] Kumar S, Stecher G, Tamura K. MEGA7: Molecular Evolutionary Genetics Analysis Version 7.0 for Bigger Datasets. Mol Biol Evol. 2016;33(7):1870–4. 10.1093/molbev/msw054 . 27004904 PMC8210823

[36] Librado P, Rozas J. DnaSP v5: a software for comprehensive analysis of DNA polymorphism data. Bioinformatics. 2009;25(11):1451–2. 10.1093/bioinformatics/btp187 19346325

[37] R-Core-Team. R: A language and environment for statistical computing. R Foundation for Statistical Computing Vienna, Austria 2016.

[38] Crean AJ, Marshall DJ. Coping with environmental uncertainty: dynamic bet hedging as a maternal effect. Philos Trans R Soc Lond B Biol Sci. 2009;364(1520):1087–96. 10.1098/rstb.2008.0237 19324613 PMC2666679

[39] Zuur AF, Leno EN, Walker NJ, Saveliev AA, Smith GM. *Mixed effects models and extensions in ecology with R* Gail M, Krickeberg K, Samet JM, Tsiatis A, Wong W, editors. Springer, New York 2009:574.

[40] Nakagawa S, Schielzeth H, O'Hara RB. A general and simple method for obtaining R^2^ from generalized linear mixed-effects models. Met Ecol Evol. 2013;4(2):133–42.

[41] Fleming IA, Gross MR. Latitudinal clines: a trade-off between egg number and size in Pacific salmon. Ecology. 1990;71(1):1–11.

[42] Marshall DJ, Allen RM, Crean AJ. The ecological and evolutionary importance of maternal effects in the sea. *Oceanography and marine biology*: *an annual review*. 2008;46:203–50.

[43] Marshall DJ, Burgess SC. Deconstructing environmental predictability: seasonality, environmental colour and the biogeography of marine life histories. Ecology letters. 2015;18(2):174–81. 10.1111/ele.12402 25534504

[44] Harrison PL. Sexual Reproduction of Scleractinian Corals In: Dubinsky Z., Stambler N. (eds) Coral Reefs: An Ecosystem in Transition. Springer, Dordrecht; 2011 p. 59–85.

[45] De Putron S, Smith S. Planula release and reproductive seasonality of the scleractinian coral *Porites astreoides* in Bermuda, a high-latitude reef. Bulletin of Marine Science. 2011;87(1):75–90.

[46] Acosta A, Zea S. Sexual reproduction of the reef coral *Montastrea cavernosa* (Scleractinia: Faviidae) in the Santa Marta area, Caribbean coast of Colombia. Marine Biology. 1997;128(1):141–8.

[47] Harii S, Omori M, Yamakawa H, Koike Y. Sexual reproduction and larval settlement of the zooxanthellate coral *Alveopora japonica eguchi* at high latitudes. Coral Reefs. 2001;20:19–23.

[48] Hwang SJ, Song JI. Sexual reproduction of the soft coral *Dendronephthya castanea* (Alcyonacea: Nephtheidae). An Cell Sys. 2012;16(2):135–44.

[49] Clarke A. Reproduction in the cold: Thorson revisited. Invertebr Repr Dev. 1992;22(1–3):175–83.

[50] Simonini R, Prevedelli D. Effects of temperature on two Mediterranean populations of *Dinophilus gyrociliatus* (Polychaeta: Dinophilidae). J Exp Mar Bio Ecol. 2003;291(1):79–93.

[51] Marshall DJ, Burgess SC. Deconstructing environmental predictability: seasonality, environmental colour and the biogeography of marine life histories. Ecol Lett. 2015;18(2):174–81. 10.1111/ele.12402 . 25534504

[52] Fernández M, Astorga A, Navarrete SA, Valdovinos C, Marquet PA. Deconstructing latitudinal species richness patterns in the ocean: does larval development hold the clue? Ecology Letters. 2009;12(7):601–11. 10.1111/j.1461-0248.2009.01315.x 19453618

[53] Seibel BA, Drazen JC. The rate of metabolism in marine animals: Environmental constraints, ecological demands and energetic opportunities. Philos Trans R Soc Lond B Biol Sci. 2007; 362(1487):2061–78. 10.1098/rstb.2007.2101 17510016 PMC2442854

[54] Padilla-Gamino JL, Alamaru A, Hedouin L, Soon SL, Portocarrero C, Bidigare RR, et al Are all eggs created equal? A case study from the Hawaiian reef building coral *Montipora capitata*. Coral Reefs. 2012;32(1):137–52.

[55] Helmuth B, Sebens K. The influence of colony morphology and orientation to flow on particle capture by the scleractinian coral *Agaricia agaricites* (Linnaeus). J Exp Mar Bio Ecol. 1993;165(2):251–78.

[56] Crook E, Cooper H, Potts D, Lambert T, Paytan A. Food availability and pCO2 impacts on planulation, juvenile survival, and calcification of the azooxanthellate scleractinian coral, Balanophyllia elegans. Biogeosciences Discussions. 2013;10(5):7761–83.

[57] Dubinsky Z, Falkowski P. Light as a source of information and energy in zooxanthellate corals *Coral Reefs*: *An Ecosystem in Transition*: Springer; 2011 p. 107–18.

[58] Waller R, Tyler P, Gage J. Reproductive ecology of the deep-sea scleractinian coral *Fungiacyathus marenzelleri* (Vaughan, 1906) in the northeast Atlantic Ocean. Coral Reefs. 2002;21(4):325–31.

[59] Woesik R. Coral communities at high latitude are not pseudopopulations: evidence of spawning at 32 N, Japan. Coral Reefs. 1995;14(2):119–20.

[60] Airi V, Gizzi F, Falini G, Levy O, Dubinsky Z, Goffredo S. Reproductive efficiency of a Mediterranean endemic zooxanthellate coral decreases with increasing temperature along a wide latitudinal gradient. PLoS One. 2014;9(3):e91792 10.1371/journal.pone.0091792 . 24618568 PMC3950289

[61] Marshall DJ, Keough MJ. The evolutionary ecology of offspring size in marine invertebrates. Adv Mar Biol. 2007;53:1–60. 10.1016/S0065-2881(07)53001-4 17936135

[62] Harrison PL. Sexual reproduction of scleractinian corals *Coral reefs*: *an ecosystem in transition*: Springer; 2011 p. 59–85.

[63] Kimura S, Tsukamoto K, Sugimoto T. A model for the larval migration of the Japanese eel: roles of the trade winds and salinity front. Mar Biol. 1994;119:185–90.

[64] Ladner JT. Hidden diversity in corals and their endosymbionts: Stanford University; 2012.

[65] Jan S, Wang J, Chern CS, Chao SY. Seasonal variation of the circulation in the Taiwan Strait. J Mar Syst. 2002;35(3):249–68.

[66] Shieh Y-T, Chen M-P. The Ancient Kuroshio Cruuent in the Okinawa Trough during the Holocene. Acta Oceanographica Taiwanica. 1995;34:73–80.

[67] Badyaev AV, Ghalambor CK. Evolution of life histories along elevational gradients: Trade-off between parental care and fecundity. Ecology. 2001;82(10):2948–60.

[68] DeBoer JA, Fontaine JJ, Chizinski CJ, Pope KL. Masked expression of life-history traits in a highly variable environment. Great Plains Research. 2015;25(1):25–38.

