# Supplementary figures and images for: Regional variability in reproductive traits of the Acropora hyacinthus species complex in the Western Pacific Region
Source: PLoS One. 2019 Jan 29;14(1):e0208605. doi: 10.1371/journal.pone.0208605 (PMC6350966; doi:10.1371/journal.pone.0208605)

S1 Fig.

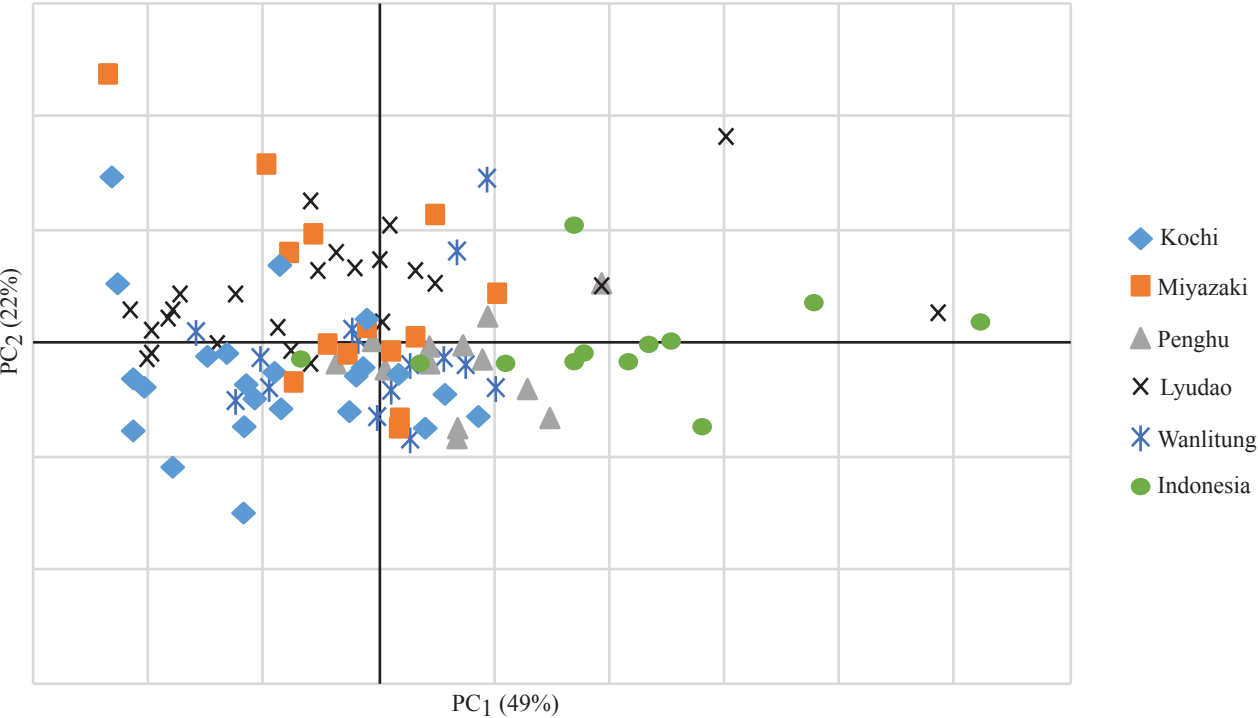

Supplement: S1 Fig — Kochi and Lyudao includes two years of data, other sites include one year of data. Traits include: egg number, median values of eggs, total testis volume and total gonad volume. (PDF) [file pone.0208605.s001.pdf]

S3 Fig.

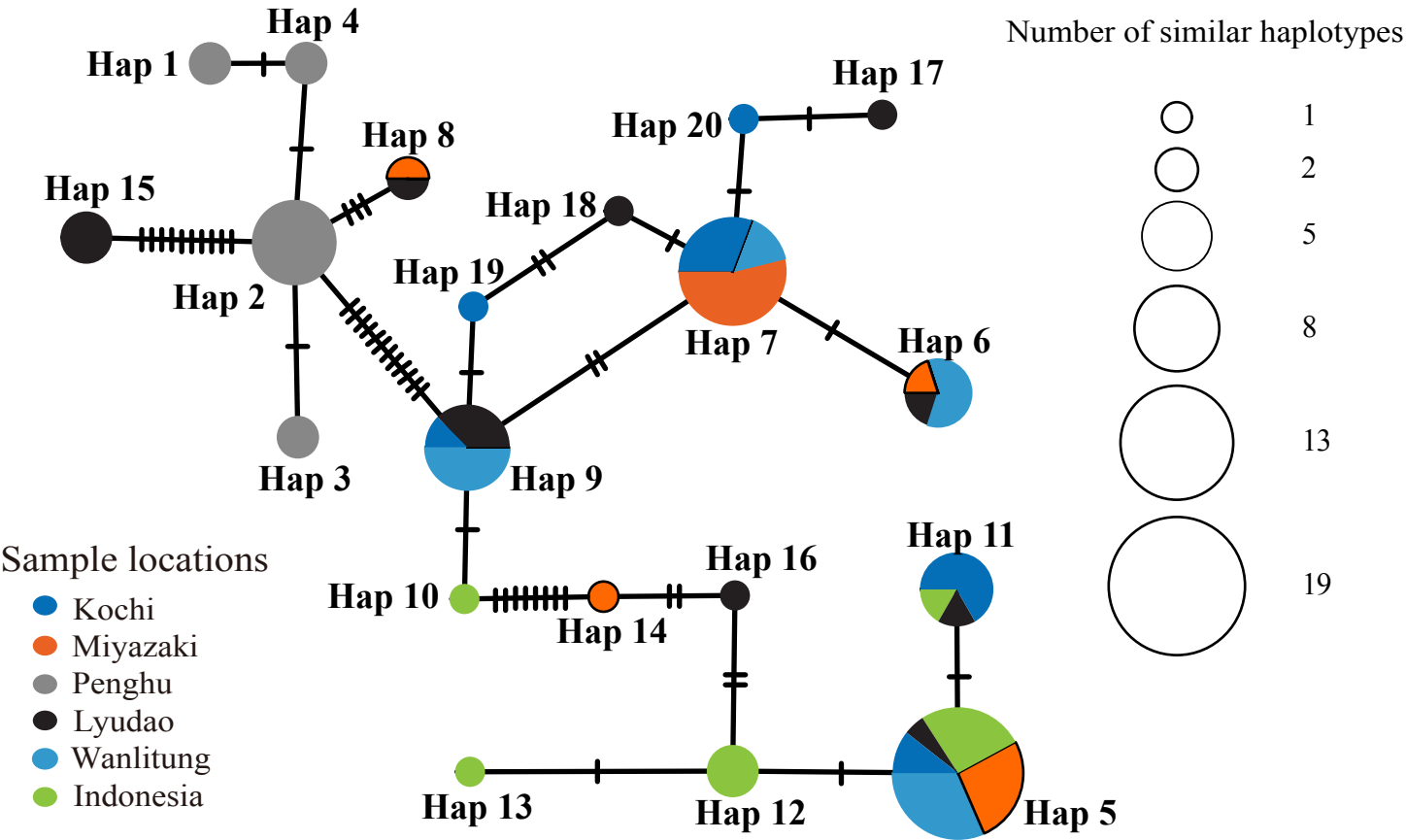

Supplement: S3 Fig — Colors indicate the sampled location. The size of each pie represents the number of similar haplotypes. (PDF) [file pone.0208605.s003.pdf]

S4 Fig.

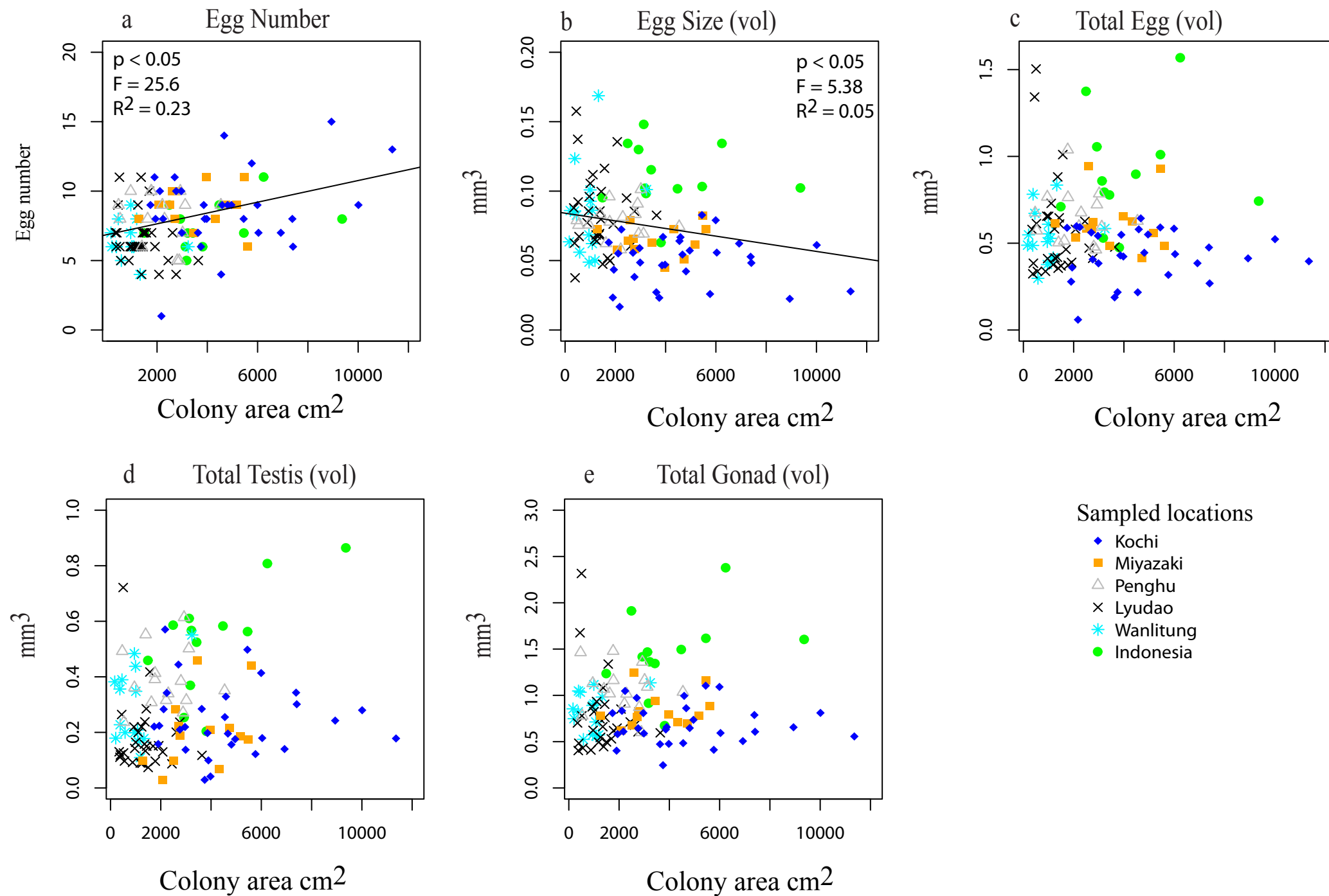

Supplement: S4 Fig — The relations between all reproductive traits and colony area at all locations using non-transformed data (n = 111). Kochi and Lyudao include two years of data. Colored icons indicate the sampled location. When significance was detected, linear regression results of p, F and R2 values are included. (PDF) [file pone.0208605.s004.pdf]

S5 Fig.

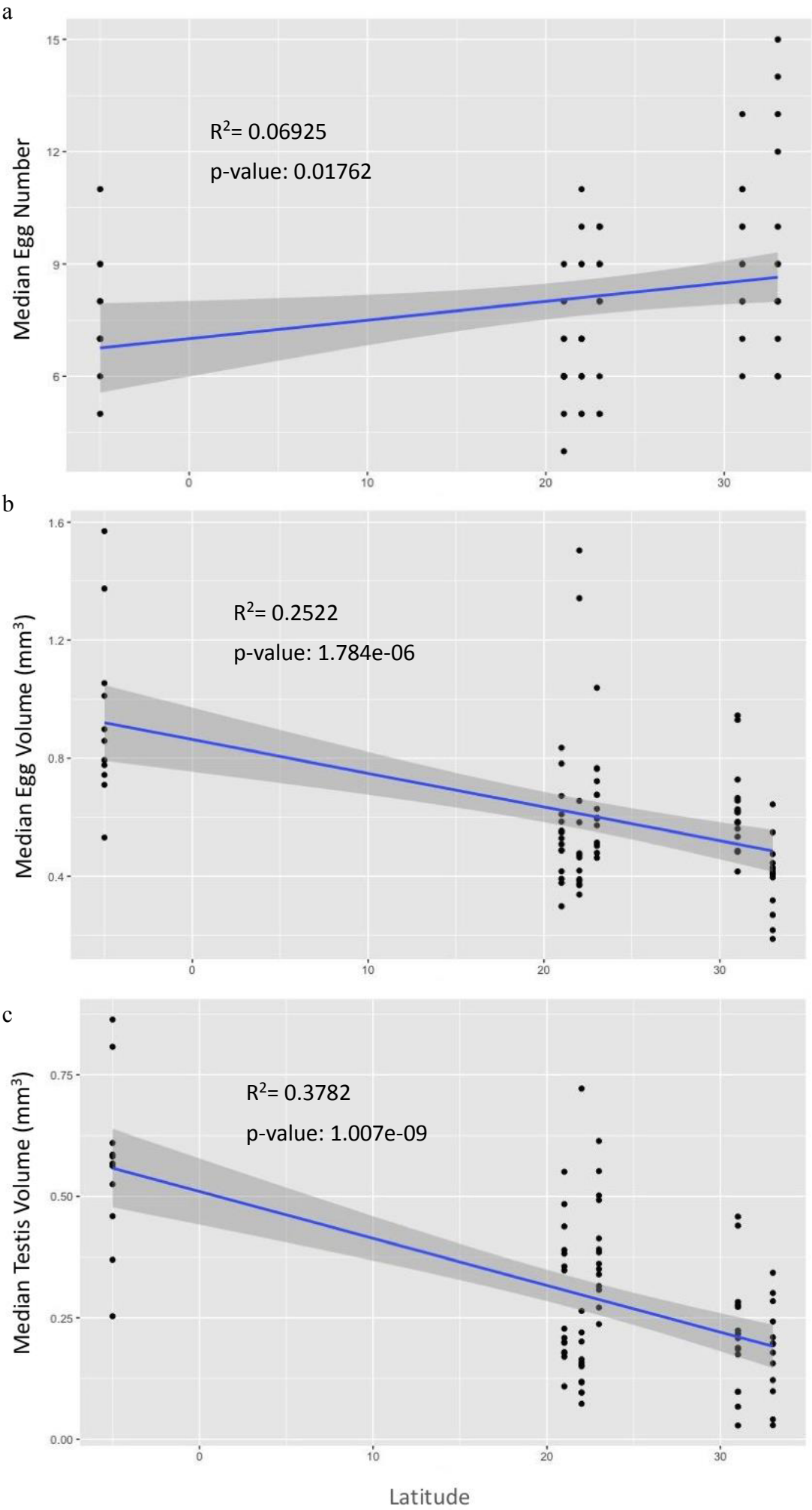

Supplement: S5 Fig — Results of the linear regression analyses are included. Shaded area = 95% confidence interval. (PDF) [file pone.0208605.s005.pdf]

S6 Fig.

a.

Kochi

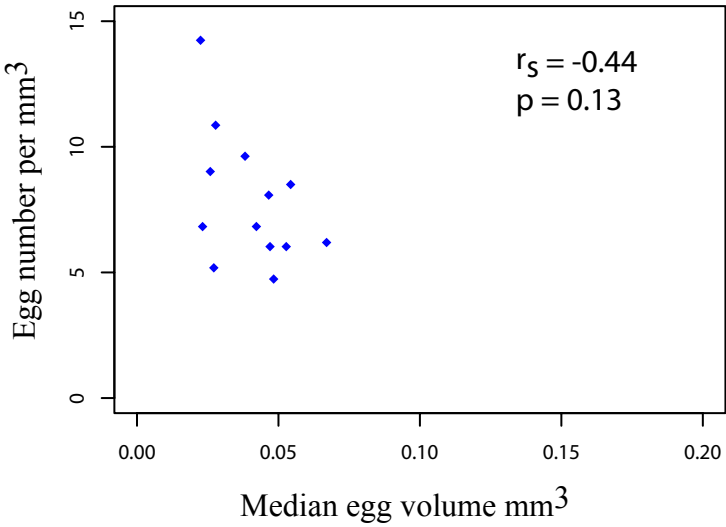

Miyazaki

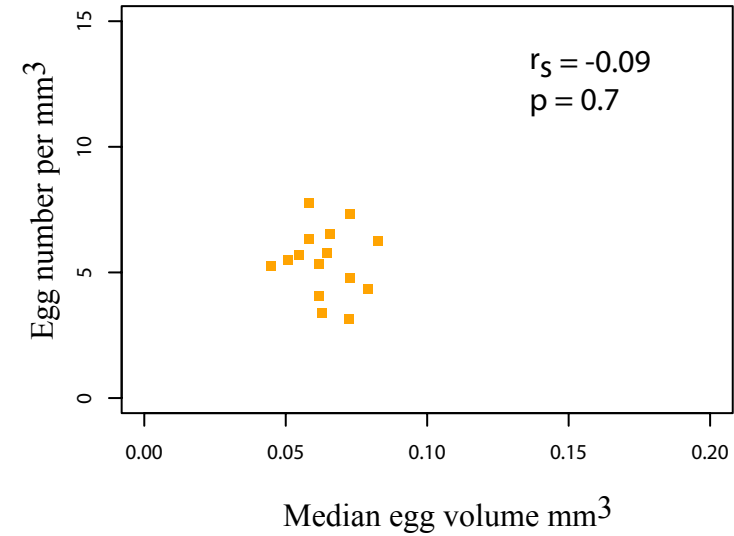

Penghu

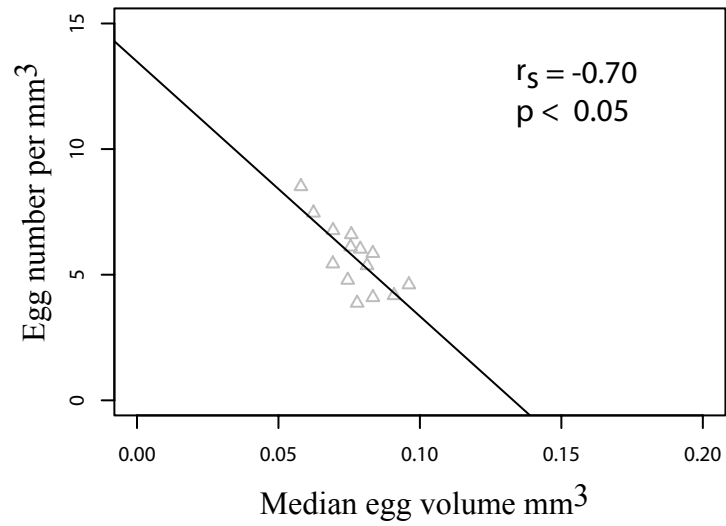

Lyudao

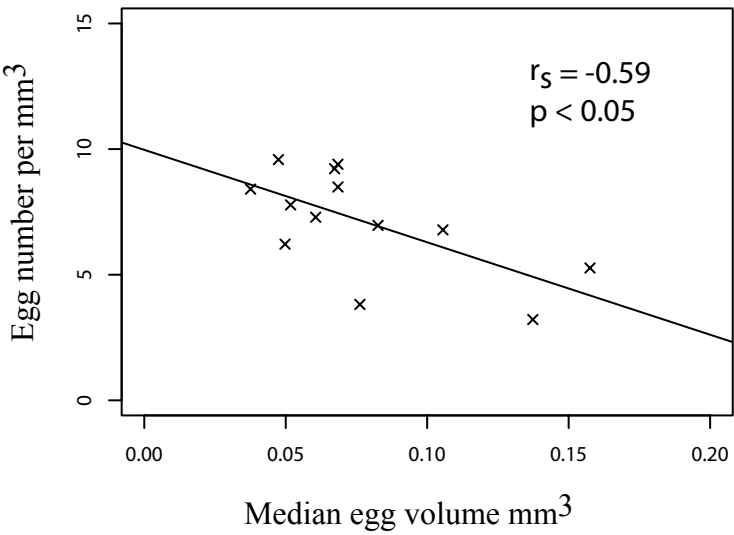

Wanlitung

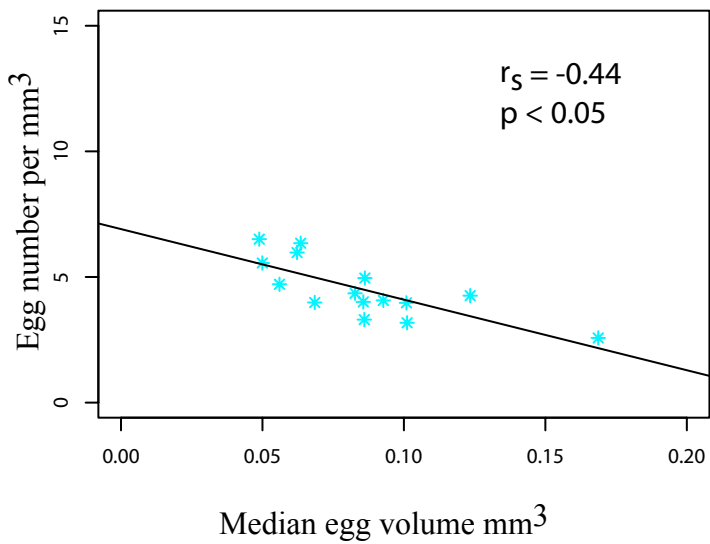

Indonesia

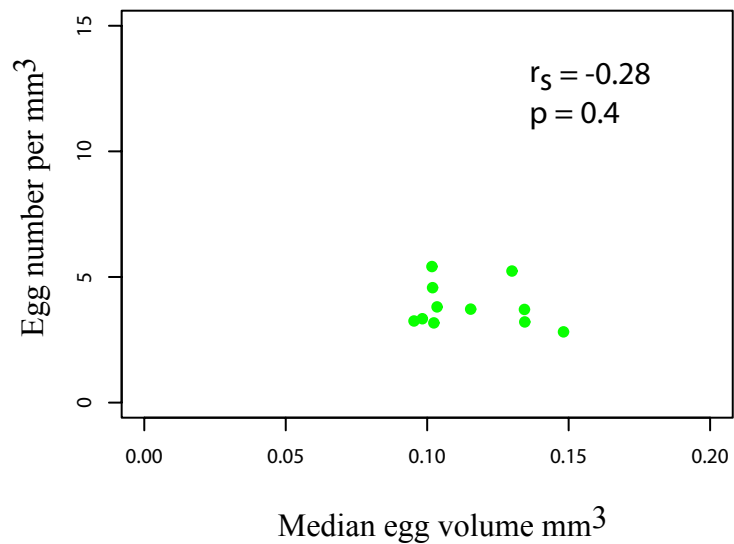

S6 Fig.

b.

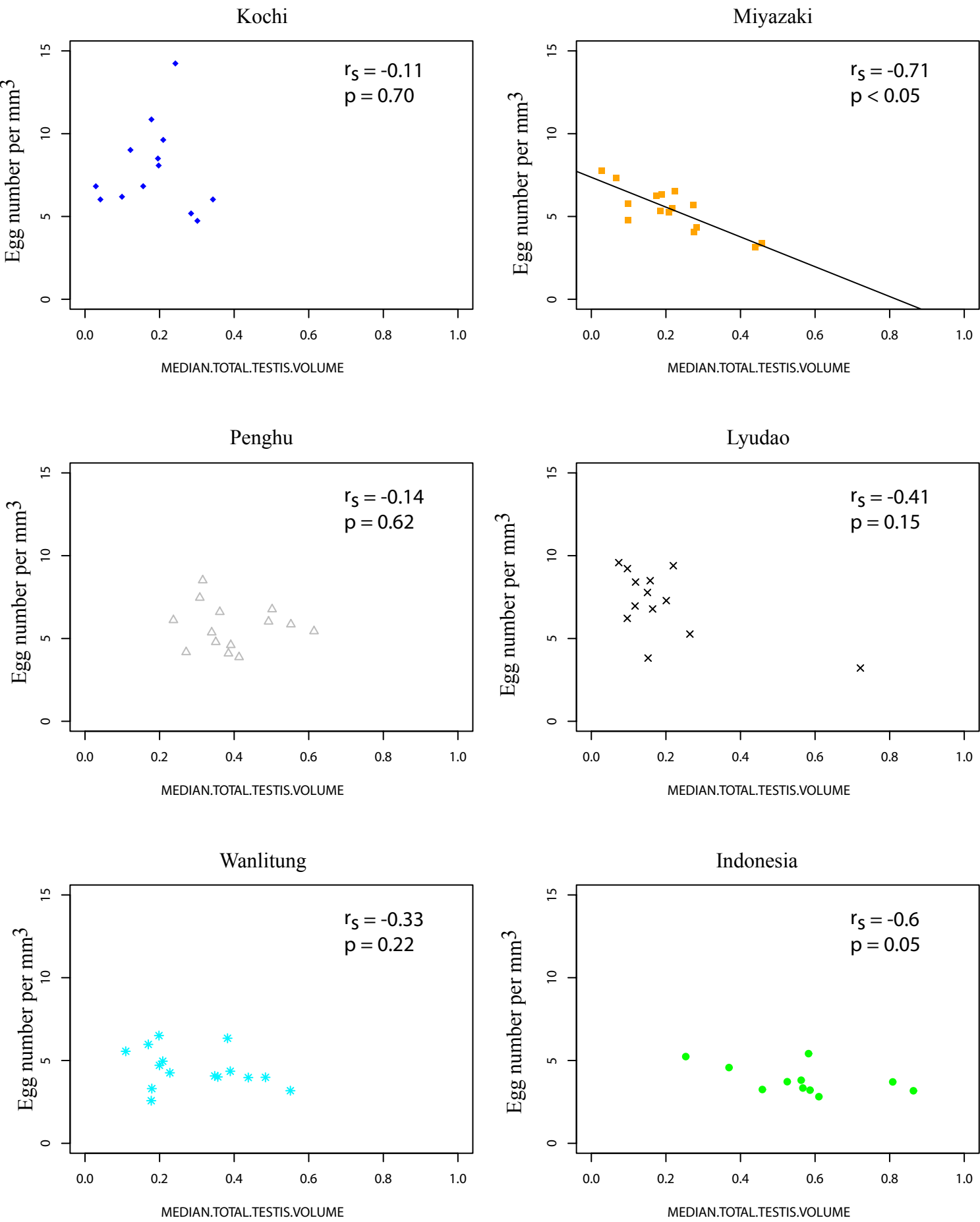

Supplement: S6 Fig — a. The relations of Egg numbers per mm3 vs median egg volume (mm3) b. The relations between Egg numbers per mm3 vs total testis volume (mm3). The results of the Spearman’s rank correlation coefficient are reported. One year of data is included per location [2014 = Indonesia (n = 12); 2015 = Kochi, Miyazaki, Penghu, Lyudao and Wanlitung (n = 15 per location)]. (PDF) [file pone.0208605.s006.pdf]

S7 Fig.

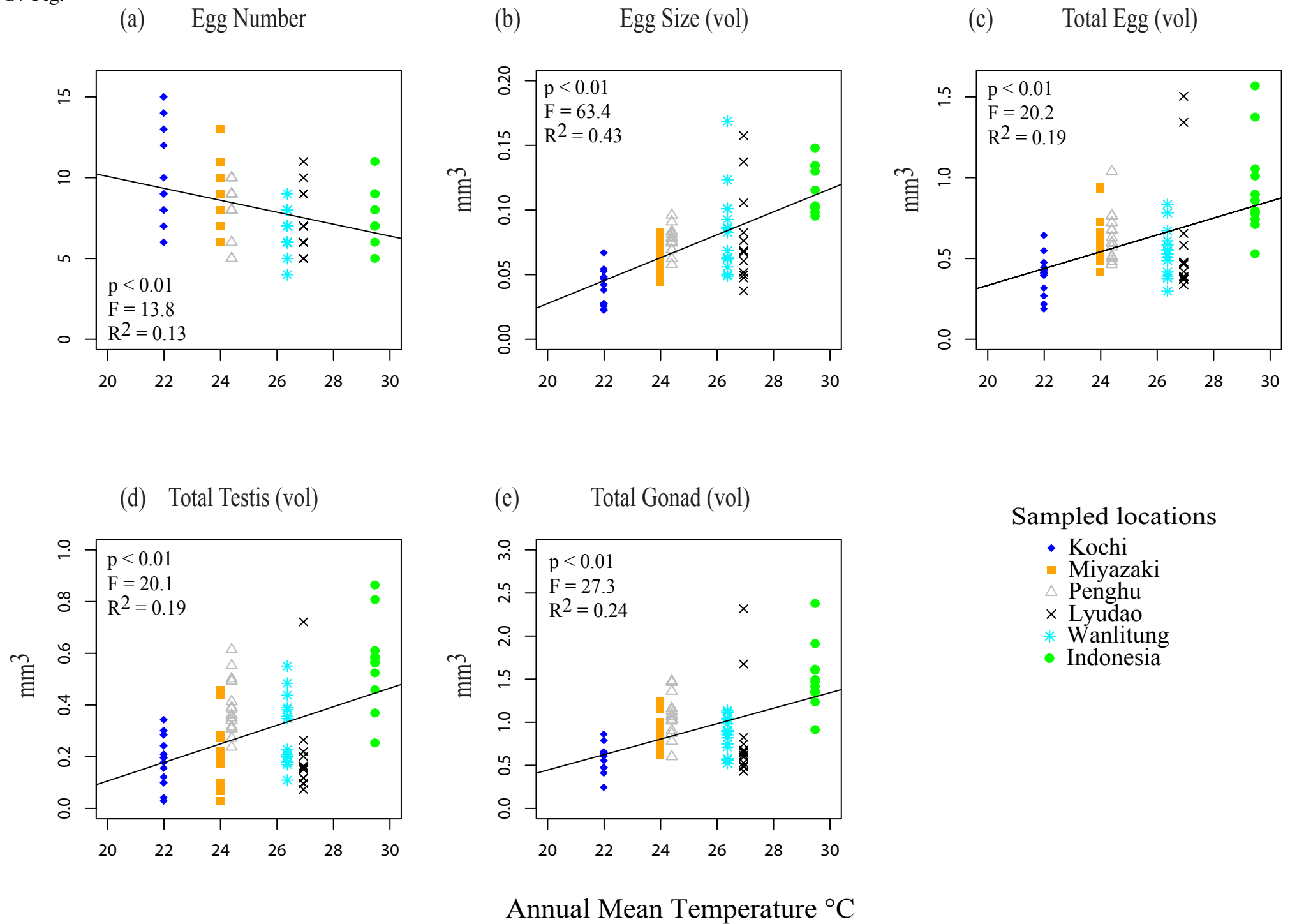

Supplement: S7 Fig — The relations between all reproductive traits and sea water temperature at all locations using non-transformed data (n = 81). Biological and physical data of the collection year were included per location (2014 = Indonesia; 2015 = Kochi, Miyazaki, Penghu, Lyudao and Wanlitung). Colored icons indicate the sampled location. When significance was detected, linear regression results of p, F and R2 values are included. (PDF) [file pone.0208605.s007.pdf]
