# Supplementary material for: Regional variability in reproductive traits of the Acropora hyacinthus species complex in the Western Pacific Region
Source: PLoS One. 2019 Jan 29;14(1):e0208605. doi: 10.1371/journal.pone.0208605 (PMC6350966; doi:10.1371/journal.pone.0208605)
Supplement: S2 Fig — The evolutionary history was inferred based on 767 bp of the putative mitochondrial control region (mtCR) of Acropora hyacinthus from the present study and at least two sequences from each of the reported cryptic lineages sequences (Hya A, B, C and D) from each clade in the phylogeny tree from Suzuki et al (2016), stored in NCBI. The previously used Acropora hyacinthus sequence from NCBI (acc. Number KF448531) was included and Isopora cuneata (acc. number AY026429) was used as outgroup. The maximum likelihood method based on the H-K-Y model and 1000 bootstrap was used. The bootstrap support group is shown next to the branches. (PDF) [file pone.0208605.s002.pdf]

S2 Fig.

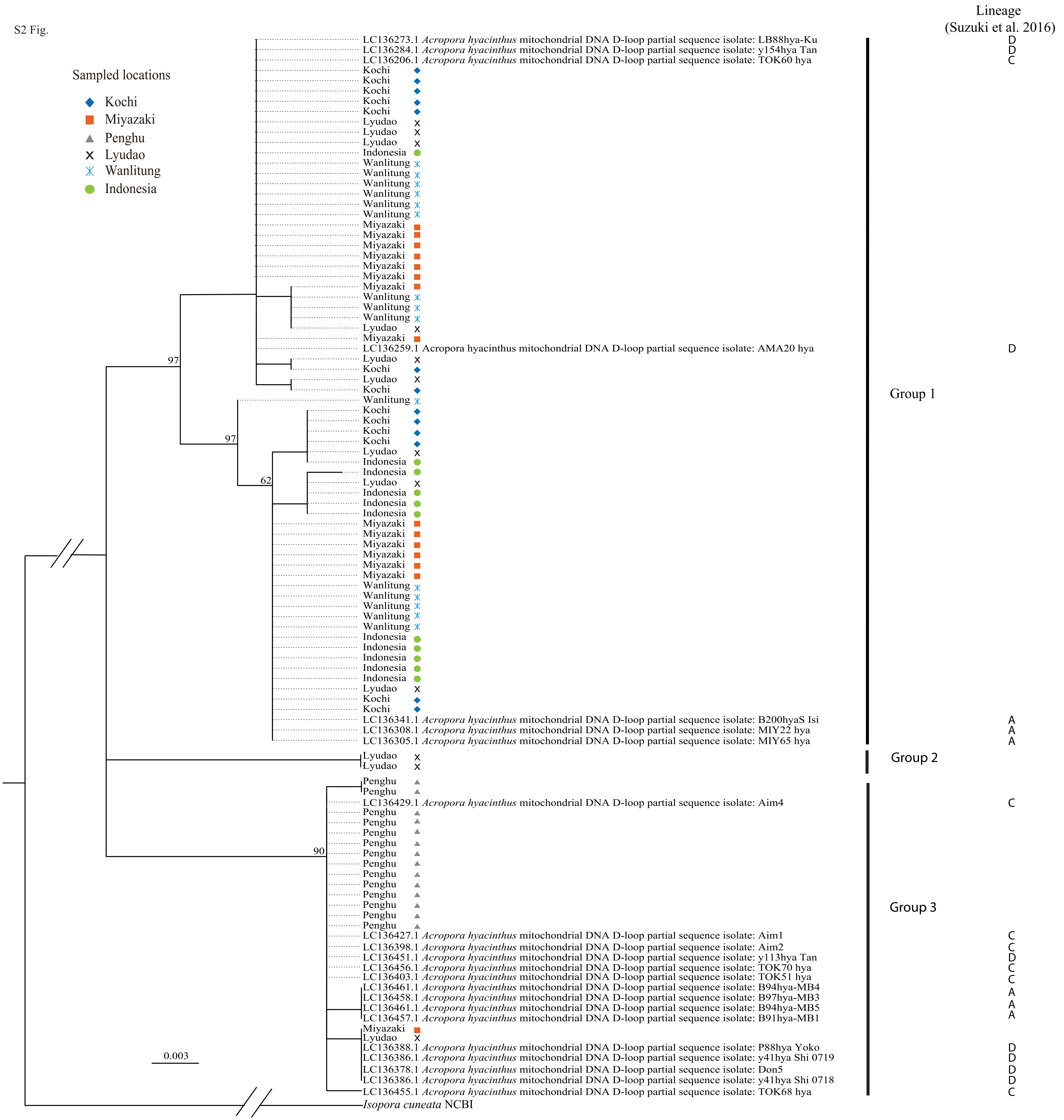
Lineage
(Suzuki et al. 2016)
Sampled locations
Kochi
Miyazaki
Penghu
Lyudao
Wanlitung
Indonesia
LC136273.1 Acropora hyacinthus mitochondrial DNA D-loop partial sequence isolate: LB88hya-Ku D
LC136284.1 Acropora hyacinthus mitochondrial DNA D-loop partial sequence isolate: y154hya Tan D
LC136206.1 Acropora hyacinthus mitochondrial DNA D-loop partial sequence isolate: TOK60 hya C
LC136259.1 Acropora hyacinthus mitochondrial DNA D-loop partial sequence isolate: AMA20 hya D
LC136341.1 Acropora hyacinthus mitochondrial DNA D-loop partial sequence isolate: B200hyaS Isi A
LC136308.1 Acropora hyacinthus mitochondrial DNA D-loop partial sequence isolate: MIY22 hya A
LC136305.1 Acropora hyacinthus mitochondrial DNA D-loop partial sequence isolate: MIY65 hya A
LC136429.1 Acropora hyacinthus mitochondrial DNA D-loop partial sequence isolate: Aim4 C
LC136427.1 Acropora hyacinthus mitochondrial DNA D-loop partial sequence isolate: Aim1 C
LC136398.1 Acropora hyacinthus mitochondrial DNA D-loop partial sequence isolate: Aim2 C
LC136451.1 Acropora hyacinthus mitochondrial DNA D-loop partial sequence isolate: y113hya Tan D
LC136456.1 Acropora hyacinthus mitochondrial DNA D-loop partial sequence isolate: TOK70 hya C
LC136403.1 Acropora hyacinthus mitochondrial DNA D-loop partial sequence isolate: TOK51 hya C
LC136461.1 Acropora hyacinthus mitochondrial DNA D-loop partial sequence isolate: B94hya-MB4 A
LC136458.1 Acropora hyacinthus mitochondrial DNA D-loop partial sequence isolate: B97hya-MB3 A
LC136461.1 Acropora hyacinthus mitochondrial DNA D-loop partial sequence isolate: B94hya-MB5 A
LC136457.1 Acropora hyacinthus mitochondrial DNA D-loop partial sequence isolate: B91hya-MB1 A
LC136388.1 Acropora hyacinthus mitochondrial DNA D-loop partial sequence isolate: P88hya Yoko D
LC136386.1 Acropora hyacinthus mitochondrial DNA D-loop partial sequence isolate: y41hya Shi 0719 D
LC136378.1 Acropora hyacinthus mitochondrial DNA D-loop partial sequence isolate: Don5 D
LC136386.1 Acropora hyacinthus mitochondrial DNA D-loop partial sequence isolate: y41hya Shi 0718 D
LC136455.1 Acropora hyacinthus mitochondrial DNA D-loop partial sequence isolate: TOK68 hya C
Isopora cuneata NCBI
Group 1
Group 2
Group 3
97
97
62
90
0.003
