# Supplementary material for: Regional variability in reproductive traits of the Acropora hyacinthus species complex in the Western Pacific Region
Source: PLoS One. 2019 Jan 29;14(1):e0208605. doi: 10.1371/journal.pone.0208605 (PMC6350966; doi:10.1371/journal.pone.0208605)

S8 Fig.

Pairwise scatterplots with Correlation coefficients

Response variable: Egg number (stdeggnumber) = egg number per mm^3

Explanatory variables: Colony area, haplotype, temperature and PAR

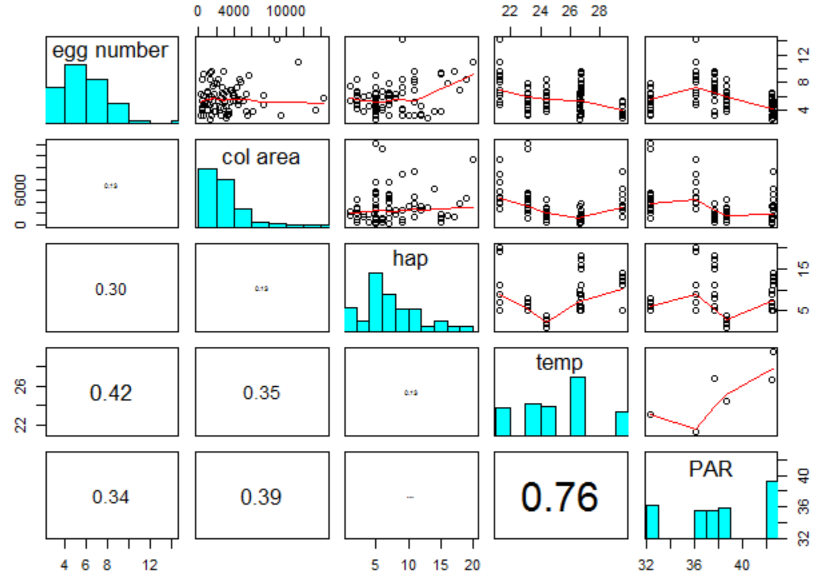

Response variable: Median egg volume

Explanatory variables: Colony area, haplotype, temperature and PAR

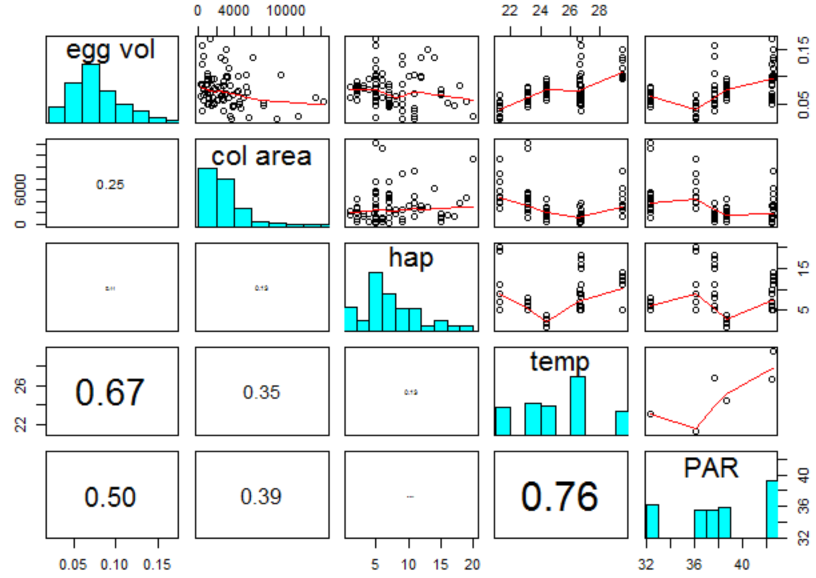

Response variable: Median total egg volume

Explanatory variables: Colony area, haplotype, temperature and PAR

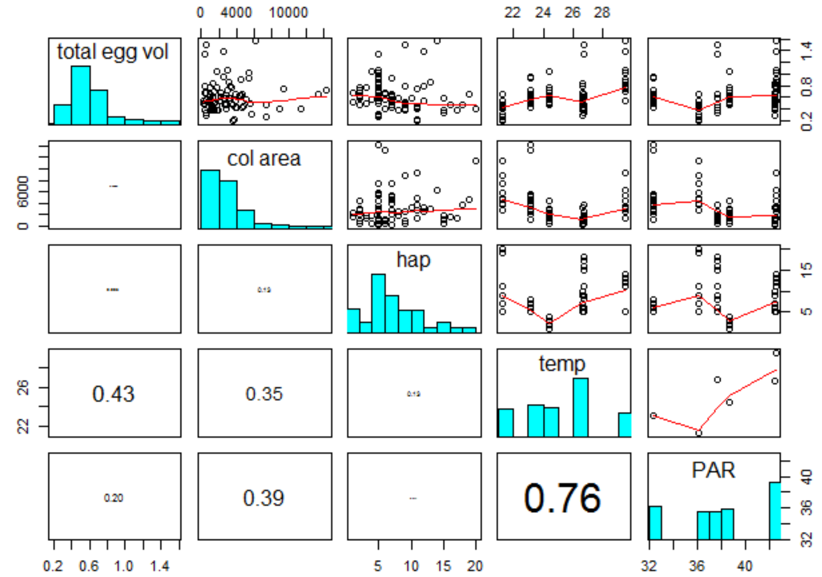

Response variable: Median total testis volume

Explanatory variables: Colony area, haplotype, temperature and PAR

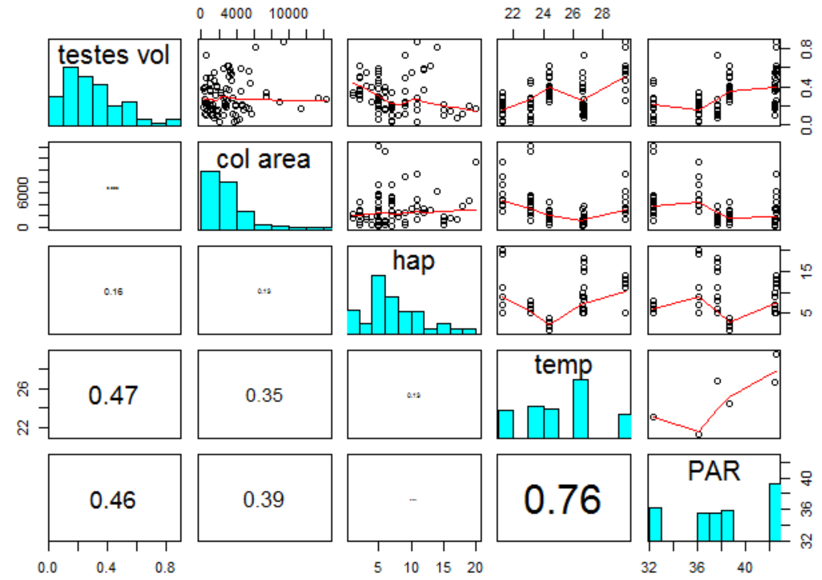

Response variable: Median total gonad volume

Explanatory variables: Colony area, haplotype, temperature and PAR

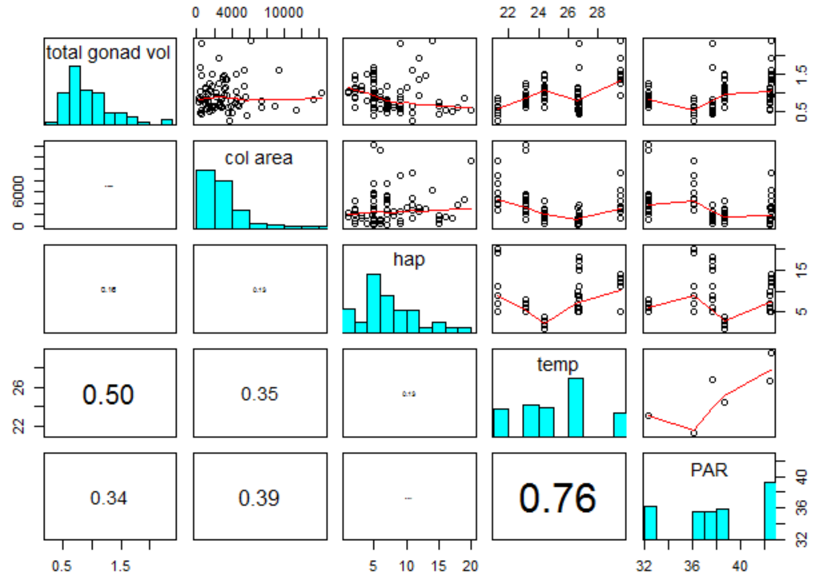

Supplement: S8 Fig — (PDF) [file pone.0208605.s008.pdf]
