# Supplementary material for: Regional variability in reproductive traits of the Acropora hyacinthus species complex in the Western Pacific Region
Source: PLoS One. 2019 Jan 29;14(1):e0208605. doi: 10.1371/journal.pone.0208605 (PMC6350966; doi:10.1371/journal.pone.0208605)
Supplement: S1 Table — Relevant information includes latitude, longitude and sample month(s) per year(s) at each location. (PDF) [file pone.0208605.s010.pdf]

S1 Table

Location	Coordinates	Sampling Dates
Kochi, Japan	33° 33’0” N<br>133° 33’0”E	July 2014/ 2015
Miyazaki, Japan	31° 54’ 0” N<br>131° 26’ 0”E	July 2015
Penghu, off West Taiwan	23° 33' 6" N<br>119° 38' 17" E	April 2015
Lyudao, off East Taiwan	22° 38' 19" N<br>121° 29' 33" E	May 2014/ 2015
Wanlitung, Southwest Taiwan	21° 59' 43" N<br>120° 42' 23" E	April 2015
Karimunjawa, Indonesia	5° 52' 0" S<br>110° 25' 58" E	March 2014
