# Supplementary material for: Regional variability in reproductive traits of the Acropora hyacinthus species complex in the Western Pacific Region
Source: PLoS One. 2019 Jan 29;14(1):e0208605. doi: 10.1371/journal.pone.0208605 (PMC6350966; doi:10.1371/journal.pone.0208605)
Supplement: S2 Table — Highest genetic distance values among locations are in boldface. (PDF) [file pone.0208605.s011.pdf]

S2 Table

Location	Miyazaki	Kochi	Penghu	Lyudao	Wanlitung	Indonesia
**Miyazaki**	0.011					
**Kochi**	0.010	0.010				
**Penghu**	**0.019**	**0.020**	0.003			
**Lyudao**	0.014	0.013	**0.017**	0.016		
**Wanlitung**	0.010	0.009	**0.019**	0.013	0.009	
**Indonesia**	0.011	0.010	**0.019**	0.014	0.010	0.004
