# Supplementary material for: Regional variability in reproductive traits of the Acropora hyacinthus species complex in the Western Pacific Region
Source: PLoS One. 2019 Jan 29;14(1):e0208605. doi: 10.1371/journal.pone.0208605 (PMC6350966; doi:10.1371/journal.pone.0208605)
Supplement: S3 Table — Summary of the one-way ANOVA results of the annual mean temperature and PAR and all measured reproductive traits across locations (Kochi and Lyudao include two years of data; the other locations include one year of data. Degree of freedom = 75. Significant p values in boldface. (PDF) [file pone.0208605.s012.pdf]

S3 Table

Variables	*F*	*p*
Mean Temperature	5.24	**2.2^{-16}**
Mean PAR	2.58	**2.2^{-16}**
Egg number per mm^3	6.70	**0.002**
Egg size vol mm^3	16.53	**0.003**
Total egg vol mm^3	8.90	**0.003**
Total testis vol mm^3	15.38	**0.001**
Total gonad vol mm^3	13.97	**0.001**
