# Supplementary material for: Regional variability in reproductive traits of the Acropora hyacinthus species complex in the Western Pacific Region
Source: PLoS One. 2019 Jan 29;14(1):e0208605. doi: 10.1371/journal.pone.0208605 (PMC6350966; doi:10.1371/journal.pone.0208605)
Supplement: S4 Table — The relationship between response variables (reproductive traits) and explanatory variables [Annual mean Temperature and Photosynthetic Available Radiation (PAR), colony area and haplotype] using linear mixed models. ‘Location’ was included as the random variable. Summary of ANOVA tests with significant values at p < 0.05 in boldface. Biological and physical data of the collection year were included per location (2014 = Indonesia; 2015 = Kochi, Miyazaki, Penghu, Lyudao and Wanlitung). (PDF) [file pone.0208605.s013.pdf]

S4 Table

Fixed variables	*Temperature*	*PAR*	*Colony area*	*Haplotype*
Response variables	p	p	p	p
Egg number per mm³	0.169	0.918	0.779	**0.045**
Egg volume	**$1.14e^{-15}$**	0.230	0.544	**0.002**
Total egg volume	**0.032**	0.246	0.430	0.134
Total testis volume	0.347	0.308	**0.015**	0.260
Total gonad volume	0.054	0.812	0.193	0.077
