# Supplementary material for: Regional variability in reproductive traits of the Acropora hyacinthus species complex in the Western Pacific Region
Source: PLoS One. 2019 Jan 29;14(1):e0208605. doi: 10.1371/journal.pone.0208605 (PMC6350966; doi:10.1371/journal.pone.0208605)
Supplement: S5 Table — Results of the blastn analysis using four sequences per lineage (HyaA, HyaB, HyaC and HyaD) from the different clades in the Suzuki et al. (2016) study downloaded as a blastn database. At least two sequences from each lineage group from the present study were used as query to perform the blastn. The maximum identity score hit from the present study sequences with the respective sequence length and those lineage ID sequences reported in the Suzuki et al. (2016) study are included in red. (PDF) [file pone.0208605.s014.pdf]

S5 Table

Query id	Phyl Group	Suzuki et al. sequence ID	Lineage ID	% identity	Al length	Mis	gap o pens	q. start	q. end	s. start	s. end	evalue	bit sco re
**Kochi_6**	1	TOK60_hya	C	100	457	0	0	1	457	106	562	0	845
**Kochi_12**	1	TOK60_hya	C	99.56	457	2	0	1	457	106	562	0	833
**Indonesia_5**	1	MIY65_hya	B	99.78	455	1	0	3	457	1	455	0	835
**Indonesia_6**	1	MIY65_hya	B	100	455	0	0	3	457	1	455	0	841
**Lyudao_2**	2	Aim4	C	98.02	455	9	0	3	457	1	455	0	791
**Lyudao_3**	2	Aim4	C	98.02	455	9	0	3	457	1	455	0	791
**Penghu_7**	3	Aim4	C	100	455	0	0	3	457	1	455	0	841
**Penghu_13**	3	Aim4	C	100	455	0	0	3	457	1	455	0	841
